# Clinical application of nanopore-targeted sequencing technology in bronchoalveolar lavage fluid from patients with pulmonary infections

**DOI:** 10.1128/spectrum.00026-24

**Published:** 2024-04-30

**Authors:** Jiayuan Ye, Kai Huang, Yaojiang Xu, Nan Chen, Yifei Tu, Jing Huang, Longfei Shao, Weiliang Kong, Dongdong Zhao, Yilian Xie

**Affiliations:** 1 Health Science Center, Ningbo University, Ningbo, Zhejiang, China; 2 Department of Infectious Diseases, Shangyu People’s Hospital Of Shaoxing, Shaoxing, Zhejiang, China; 3 Department of General Medicine, The First Affiliated Hospital of Ningbo University, Ningbo, Zhejiang, China; 4 Department of Radiology, Shangyu People’s Hospital Of Shaoxing, Shaoxing, Zhejiang, China; 5 Department of Respiratory, Shangyu People’s Hospital Of Shaoxing, Shaoxing, Zhejiang, China; 6 Key Laboratory of Digital Technology in Medical Diagnostics of Zhejiang Province, Dian Diagnostics Group Co., Ltd., Hangzhou, Zhejiang, China; 7 Department of Respiratory and Critical Care Medicine, The First Affiliated Hospital of Ningbo University, Ningbo, Zhejiang, China; 8 Department of Infectious Diseases, Sir Run Run Shaw Hospital, Zhejiang University School of Medicine, Hangzhou, Zhejiang, China; 9 Department of Infectious Diseases, The First Affiliated Hospital of Ningbo University, Ningbo, Zhejiang, China; Institut National de Santé Publique du Québec, Sainte-Anne-de-Bellevue, Canada

**Keywords:** pulmonary infection, bronchoalveolar lavage fluid, pathogen detection, nanopore, clinical diagnosis

## Abstract

The rapid and effective identification of pathogens in patients with pulmonary infections has posed a persistent challenge in medicine, with conventional microbiological tests (CMTs) proving time-consuming and less sensitive, hindering early diagnosis of respiratory infections. While there has been some research on the clinical performance of targeted sequencing technologies, limited focus has been directed toward bronchoalveolar lavage fluid (BALF). This study primarily evaluates the pathogen detection capabilities of nanopore-targeted sequencing (NTS) in BALF, providing a comprehensive analysis. The retrospective study, spanning from January 2022 to November 2023, includes 223 patients exclusively sourced from a single center. We conducted a detailed comparative analysis among NTS, targeted next-generation sequencing (tNGS), and CMTs. Initially, we compared the detection capabilities of NTS and tNGS and found no significant differences in their sensitivity and specificity. Specifically, we observed that the sensitivity of NTS was significantly higher than that of CMTs (74.83% vs 33.11%, *P* < 0.001). Furthermore, NTS exhibited a higher positivity rate in common pulmonary infections (62.88% vs. 23.48%) and in clinically suspected tuberculosis patients compared to CMTs (87.18% vs. 48.72%). Additionally, NTS showed less susceptibility to antibiotic interference, indicating a more sensitive detection capability, especially in detecting fastidious organisms. It complements GeneXpert in tuberculosis diagnosis and offers excellent advantages in identifying pathogens challenging for CMTs, such as non-tuberculous mycobacteria and viruses. Moreover, NTS significantly shortens the reporting time and is only a quarter of the cost of metagenomic next-generation sequencing. Clearly, NTS can facilitate faster and more cost-effective early diagnosis of respiratory infections.

**IMPORTANCE:**

This study holds paramount significance in advancing the field of respiratory infection diagnostics. By assessing the pathogen detection capabilities in bronchoalveolar lavage fluid (BALF) of patients with pulmonary infections, we illuminate the promising potential of nanopore-targeted sequencing (NTS). The findings underscore NTS as a comparable yet distinct alternative to traditional methods like comprehensive conventional microbiological tests (CMTs). Notably, NTS demonstrates a pivotal edge, expanding the spectrum of identified pathogens, particularly excelling in the detection of challenging entities like non-tuberculous mycobacteria and viruses. The study also highlights the complementary role of NTS alongside GeneXpert in the identification of tuberculosis, providing a comprehensive overview of the diagnostic landscape for respiratory infections. This insight carries significant implications for clinicians seeking rapid, cost-effective, and accurate diagnostic tools in the realm of pulmonary infections.

## INTRODUCTION

Infectious diseases, with their profound impact on socio-economic development (1) and their role as a leading cause of human mortality, remain a significant global challenge. Among these diseases, pulmonary infections are particularly noteworthy due to their rapid onset, severity, and numerous complications, making them the most fatal among all infectious diseases (2). Lung infections caused by bacteria, viruses, or fungi rank as the third leading cause of overall life expectancy loss (3). According to the Global Burden of Disease Study, respiratory infectious diseases caused a global life expectancy loss of 1.29 years in 2017 (4). Specific infections, such as those induced by mycobacteria leading to chronic infectious diseases, pose a serious threat to human health. The mortality rate of tuberculosis varies from 7% to 35% globally, with each country facing a unique situation (5). Early detection and control of infection sources can effectively interrupt the transmission of tuberculosis, contributing to the alleviation of the disease burden (6).

Despite the availability of various diagnostic methods, achieving rapid and accurate identification of pathogenic agents remains a formidable challenge. Traditional pathogen identification relies on conventional culture, polymerase chain reaction (PCR) detection, and antigen/antibody immunological methods (7). These approaches are time-consuming with low positive detection rates, falling short of meeting clinical demands, particularly in the time-intensive culturing of mycobacteria and fungi. Conventional pathogen detection approaches yield the identification of pathogens in only 38% of pneumonia cases (8). Furthermore, traditional culture outcomes are susceptible to the influence of prior empiric infection treatments and sampling specifications, markedly diminishing diagnostic sensitivity (9, 10). Research indicates a mere 0–14% detection rate in blood cultures, even among patients with severe pulmonary infections (11, 12). Even with advanced technologies like MTB GeneXpert designed for tuberculosis detection, whose sensitivity can reach up to 74.5% (13), its functionality is relatively singular and cannot meet clinical demands due to the limitations of single-target detection. Certain methods, such as PCR testing and antigen/antibody immunological approaches, must be grounded in known genetic sequences or target proteins of the pathogens (14). Although PCR-based single or multiplex nucleic acid detection technologies provide rapid and sensitive pathogen detection, their coverage is limited, detecting only 1–10 pathogens in a single test (15).

Metagenomic next-generation sequencing (mNGS) is a novel and promising approach that integrates high-throughput sequencing with bioinformatics analysis, offering a broad range of detectable pathogens (16). However, challenges such as extended detection cycles and high costs hinder the development of mNGS. In response to these challenges, a more rapid and cost-effective technology, targeted next-generation sequencing (tNGS), has emerged for achieving an early diagnosis of respiratory infections. tNGS has been used to identify pathogens in cases of respiratory infections or mycobacterial infections, providing sufficient accuracy and significantly higher sensitivity in pathogen detection (13, 17). Additionally, nanopore-targeted sequencing (NTS), a targeted third-generation sequencing technology that combines ultra-multiplex PCR amplification with high-throughput nanopore sequencing, has been developed and shows promising clinical applications in the diagnosis of lower respiratory tract infectious diseases (18). This technique directly reads base information by detecting current changes when nucleic acid molecules pass through nanopores (19). With its rapid sequencing speed (exceeding 400 bp/s) and longer read lengths (average read length exceeding 1,000 bp), nanopore sequencing significantly enhances sequencing efficiency, enabling precise detection (19). Early investigations suggest that NTS shows heightened sensitivity compared to MTB GeneXpert, particularly in detecting MTB. Furthermore, NTS and mNGS demonstrate comparable sensitivity in respiratory sample detection, yet NTS showcases superior specificity compared to mNGS. Importantly, NTS emerges as a promising solution to overcome the constraints associated with culture, PCR, and mNGS (20).

Herein, we undertook a retrospective analysis of clinical data encompassing 223 patients suspected of pulmonary infections admitted to a singular medical center in China from January 2022 to November 2023. We compared the sensitivity and specificity of tNGS and NTS technologies with CMTs in identifying pathogens. Additionally, we evaluated the diagnostic utility of tNGS and NTS in bronchoalveolar lavage fluid (BALF) from individuals presenting with pulmonary infections.

## MATERIALS AND METHODS

### Study center and collection of samples

This study was conducted at Shangyu People’s Hospital in Shaoxing, Zhejiang Province, China, from January 2022 to November 2023. A total of 223 patients with pulmonary infections were included. All patients underwent bronchoscopy, and BALF was collected. The study received approval from the Ethics Committee of Shangyu People’s Hospital, Shaoxing, and informed consent was obtained from all patients or authorized family members.

### Inclusion criteria

The inclusion criteria are as follows: (i) Patients of any age or gender. (ii) Specific diagnostic criteria for pulmonary infections include the presence of new or worsening focal or diffuse infiltrates on chest X-ray or computed tomography (CT), combined with clinical manifestations such as new-onset fever, cough, increased sputum production, shortness of breath, and hemoptysis. (iii) Collection of BALF. (iv) Patients who have provided signed informed consent.

### Exclusion criteria

1. Cases with incomplete data.
2. Sample-related issues: inadequate sample volume or samples not meeting standard requirements.

### Subgroup design

**Fig 1:**
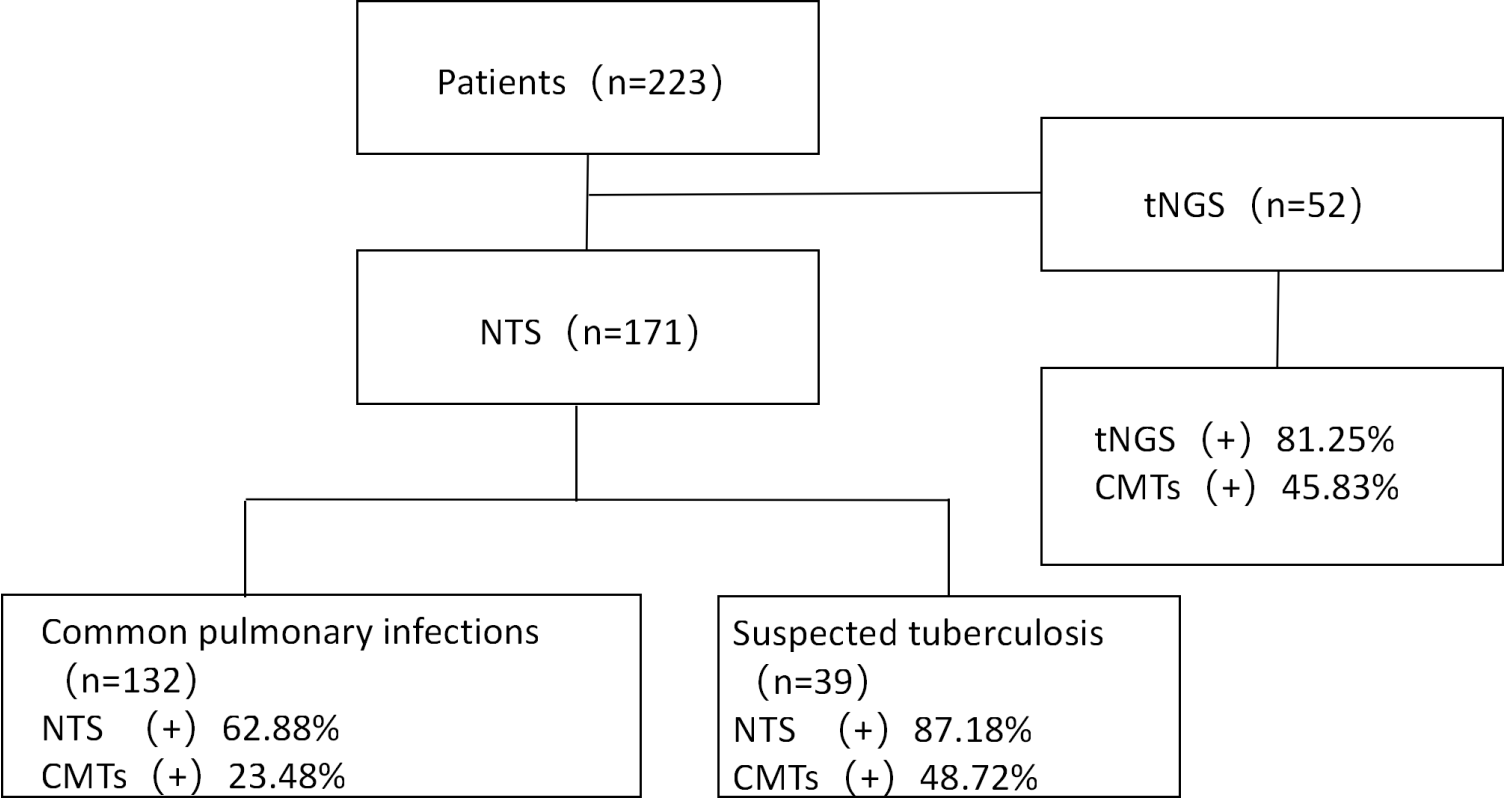
Flowchart of this study. A total of 223 patients were enrolled and divided into the tNGS group and NTS group (encompassing common pulmonary infections and suspected tuberculosis). Abbreviations: tNGS, targeted second-generation sequencing; NTS, targeted third-generation sequencing; CMT, conventional microbial testing.

According to the different testing methods received, 223 patients were stratified into two main groups, with 56 patients in the tNGS group and 171 patients in the NTS group. The NTS group is further divided into 39 patients in the suspected tuberculosis group [clinical diagnostic criteria for tuberculosis refer to the WS 288-2017 Tuberculosis Diagnostic Criteria (21)], and 132 cases of common pulmonary infections (Fig. 1).

### Sample collection and laboratory testing

All patients underwent bronchoscopy examination. BALF was selected based on lesion locations identified through chest CT imaging, and further testing was conducted on BALF using CMTs, tNGS, NTS, and others.

### tNGS and NTS testing

BALF samples (>5 mL) were collected and promptly transported on dry ice to Hangzhou KingMed Diagnostics Laboratory for tNGS and to Hangzhou Dean Medical Laboratory for NTS. Detailed descriptions of the testing procedures conducted by both companies can be found in the accompanying documents (Supplementary 1 and Supplementary 2).

### Criteria for positive tNGS results

A sample was considered to pass quality control when the minimum raw reads exceeded 50k, the internal reference gene amplification reads were at least 200, and the Q30 value exceeded 75%. For results meeting the quality criteria, if the normalized reads for at least one target of the pathogen were ≥20, the pathogen was considered positive; otherwise, it was deemed negative.

### Criteria for positive NTS results

A sample was deemed to have passed quality control when the data volume reached 30 Mb, and the number of sequences detected for the internal control (*Lactococcus lactis*) exceeded 100. Species information for each sequencing sequence was determined by aligning it with database sequences using the highly reliable local alignment search tool (BLAST). If over 80% of the database sequences in the BLAST results belonged to a specific species, and the alignment coverage was not less than 85%, the species information was assigned to the sequencing sequence. The focus of the analysis was the percentage of reads related to specific species within the same batch of samples. Any proportion below 0.5% was considered the result of cross-contamination within the batch and could not be interpreted as a positive result (20).

### Negative control

Examine the species results of the negative control sample. If a species is detected in both the negative control and test samples, but the number of reads for that species in the test sample exceeds five times that of the negative control, then retain that species. Otherwise, it is considered not to be a pathogenic microorganism and is discarded.

### CMTs testing

Routine microbiological tests were performed on BALF samples. These included Gram staining, acid-fast bacilli smear, bacterial culture, mycobacterial culture, and fungal culture. Additionally, GeneXpert was employed. These tests were complemented by serum G test, GM test, and cryptococcal antigen detection. PCR testing for SARS-CoV-2 and influenza A and B was conducted from throat swabs. Furthermore, *Mycoplasma pneumoniae*, *Chlamydia pneumoniae*, respiratory syncytial virus, adenovirus, cytomegalovirus, and Epstein-Barr virus antigen/antibody immunoassays were performed. The specific methods can be found in supplementary 3.

### Establishment of the final clinical diagnosis

The final clinical diagnosis of each participant enrolled in the study served as the benchmark for assessing the sensitivity and specificity of the detection methods. This diagnosis was established at the time of patient discharge, following consultations among two infectious disease physicians and one radiologist from the medical team. In cases where a unanimous decision couldn't be reached, the research team organized a meeting with three experts to achieve a conclusive diagnosis. The evaluation took into account the patient’s clinical characteristics, findings from conventional pathogen detection, tNGS, NTS, pathology data, outcomes, and other relevant factors to ensure a comprehensive assessment.

### Statistical analysis

Statistical analysis was conducted using SPSS 25.0 software (IBM Corporation, Chicago, IL, USA). Clinical comprehensive diagnosis served as the reference standard. Pearson’s *χ*^2^ test or Fisher’s exact test was employed to compare the sensitivity and specificity among tNGS, NTS, and CMTs. All tests were two-sided, and a *P* value < 0.05 was considered statistically significant. Venn diagram was performed using R (version 4.3.0). Heat map was performed using GraphPad Prism 10.

## RESULTS

### Basic characteristics of enrolled patients

**Fig 2:**
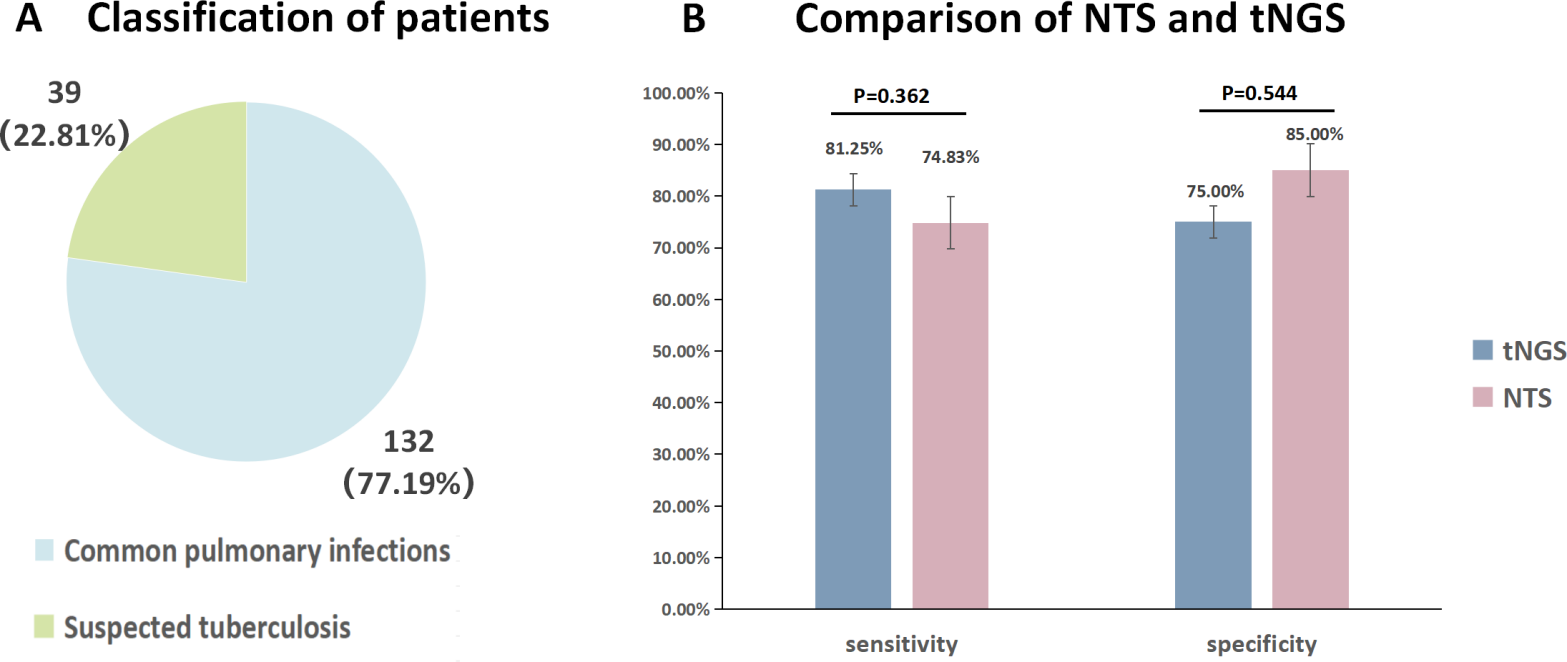
(**A**) The classification of patients in the NTS group is further divided into suspected tuberculosis and common pulmonary infections. (**B**) Comparison of sensitivity and specificity between NTS (*N* = 171) and tNGS (*N* = 52) to assess the diagnostic capabilities of tNGS and NTS using *χ*^2^ statistics.

In this study, we enrolled 223 patients from a single hospital between January 2022 and December 2023. Among them, 56 underwent tNGS, while 171 underwent NTS. Sensitivity and specificity analyses were conducted separately for tNGS and NTS patients, with a more detailed analysis performed specifically for the 171 NTS patients. Key features of the NTS patient group are presented below. The average age of these patients was 54 years (14–85 years), with a male-to-female ratio of 1.12. Bronchiectasis was the predominant comorbidity (*n* = 22). After a thorough evaluation, NTS patients were stratified into the infectious diseases (ID) group (151 cases, 88.30%) and the non-infectious diseases (NID) group (20 cases, 11.69%). Furthermore, patients were categorized based on chest CT images and clinical features into common pulmonary infections (132 cases, 77.19%) and suspected tuberculosis (39 cases, 22.81%) (Fig. 2).

### The diagnostic performance of tNGS and NTS

The pathogen detection spectrum of NTS included 354 pathogens, while tNGS detected 198 pathogens. We conducted a comparative analysis on the overall sensitivity and specificity of tNGS (52 patients) and NTS (171 patients), revealing no significant difference in sensitivity (81.25% vs 74.83%, *P* = 0.362) and specificity (75.00% vs 85.00%, *P* = 0.544) (Fig. 2).

### The diagnostic performance of NTS and CMTs

**Fig 3:**
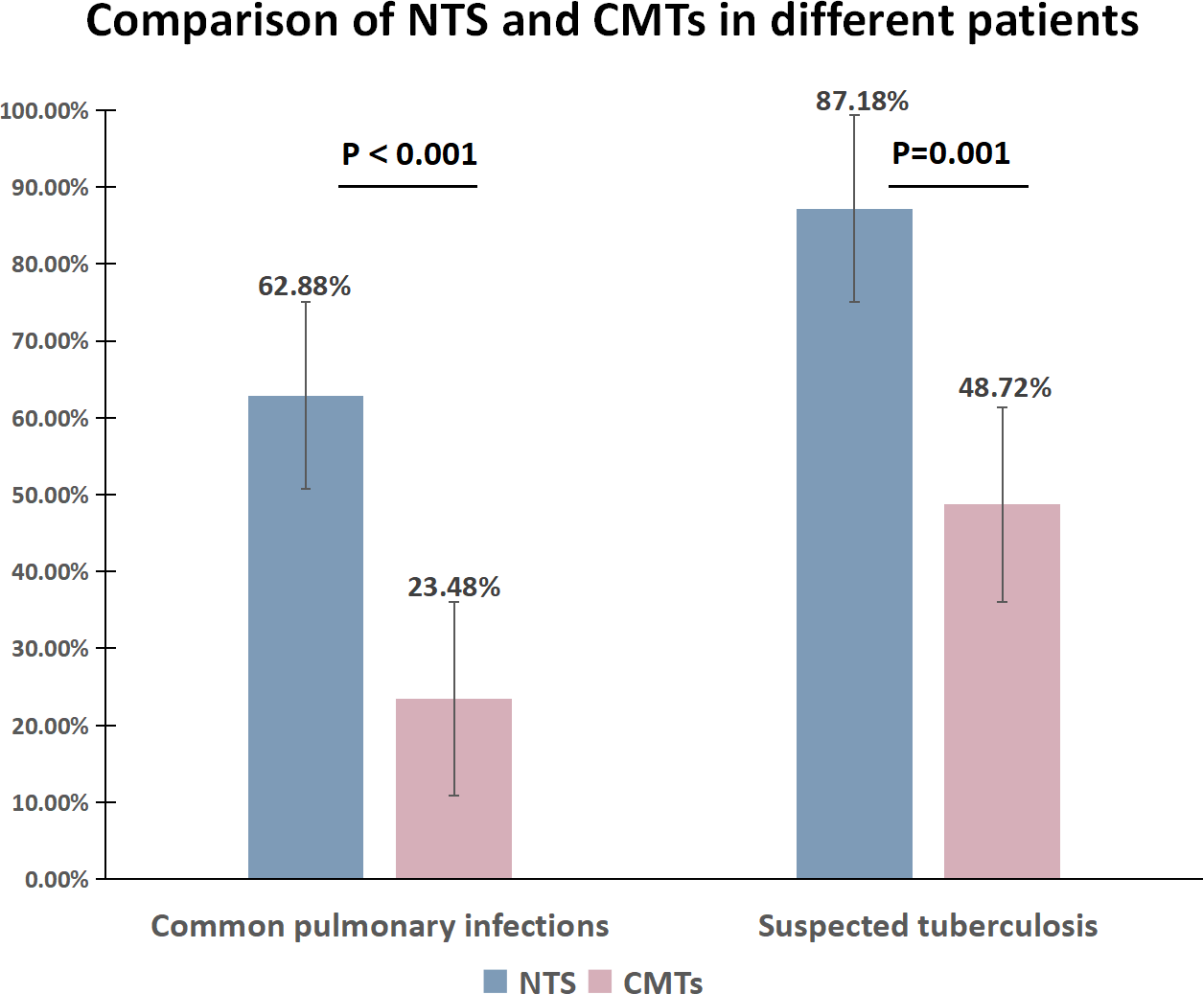
Comparison of performance between methods NTS and CMTs in detecting pathogens in BALF.

**Fig 4:**
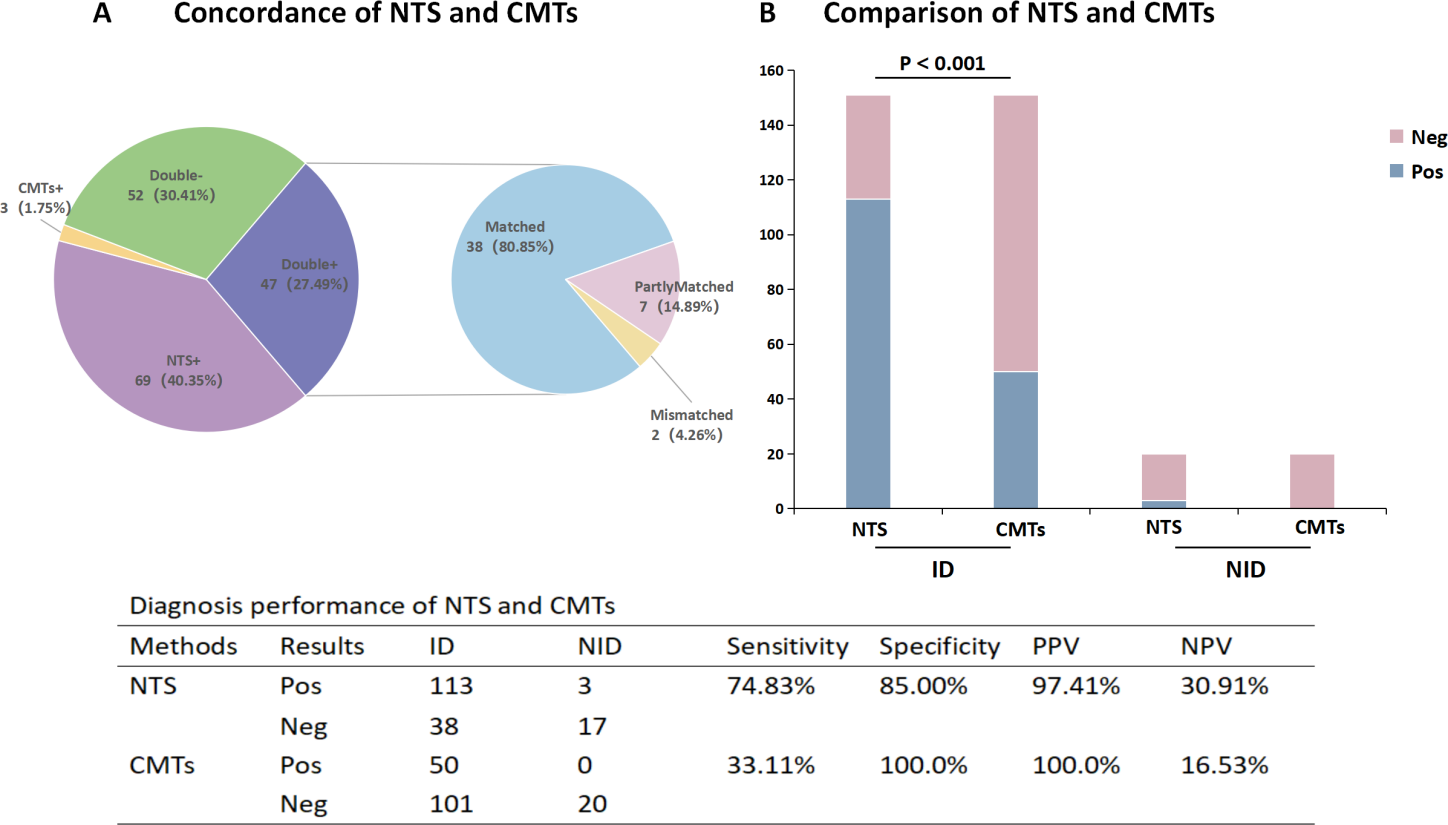
The concordance and diagnostic ability of NTS and CMTs. (**A**) Concordance of NTS and CMTs. (**B**) Comparison of diagnostic ability between NTS and CMTs. Abbreviation: ID: infectious disease; NID: non-infectious disease; Pos, positive; Neg, negative; PPV, positive predictive value; NPV, negative predictive value. Mismatch: Positive results in NTS do not match positive results in CMTs.

The positivity rate of NTS was significantly higher than that of CMTs in both common pulmonary infections (62.88% vs 23.48%, *P* < 0.001) and the suspected tuberculosis group (87.18% vs 48.72%, *P* = 0.001) (Fig. 3). Additionally, 47 patients (27.49%) exhibited positive results for both NTS and CMTs, while 52 patients (30.41%) showed negative results for both. Among the 47 cases with double-positive results, 38 cases (80.85%) demonstrated complete concordance, 7 cases (14.89%) exhibited partial concordance, and 2 cases (4.26%) showed discordance (Fig. 4).

### The diagnostic performance of tNGS and CMTs

**Fig 5:**
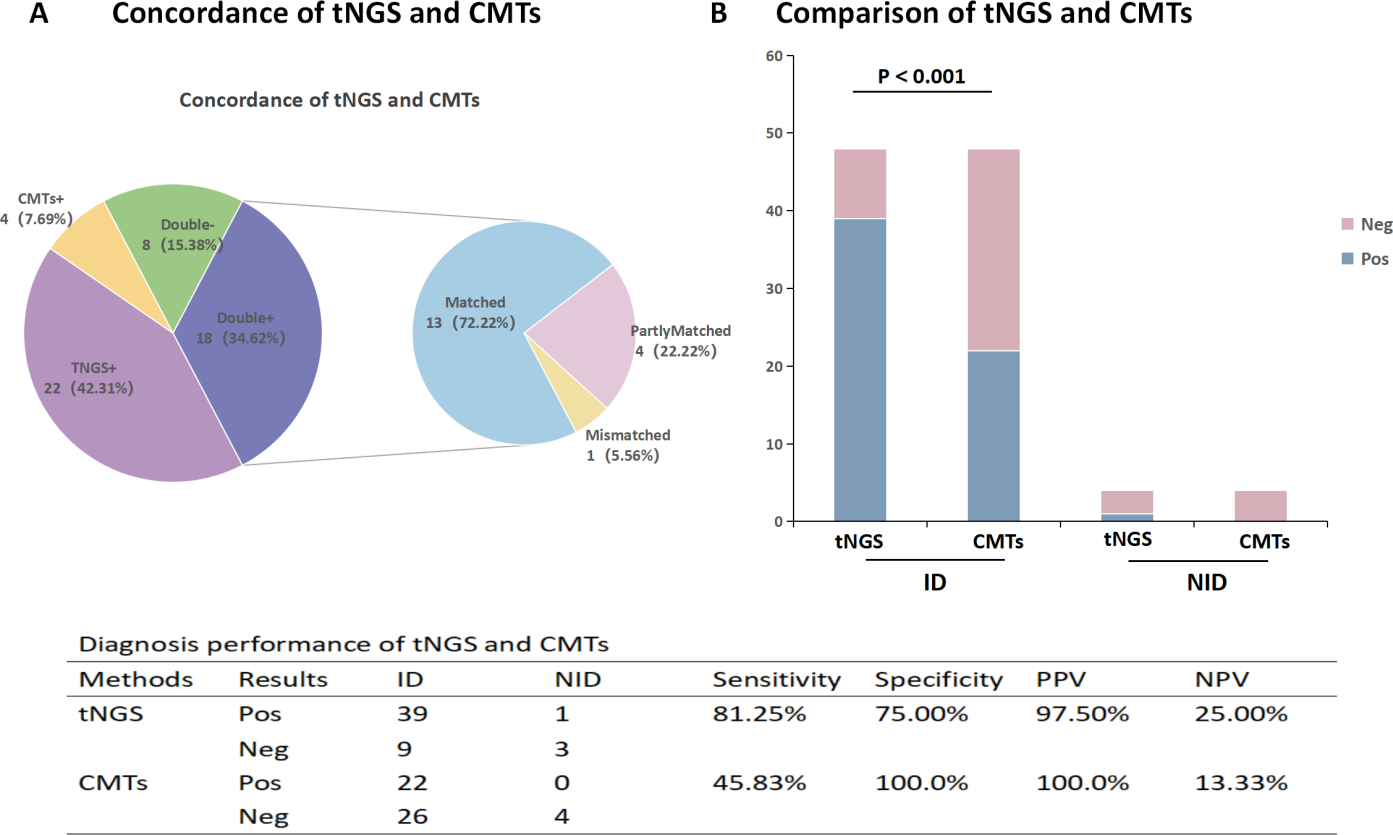
The concordance and diagnostic ability of tNGS and CMTs. (**A**) Concordance of tNGS and CMTs. (**B**) Comparison of diagnostic ability between tNGS and CMTs. Abbreviation: ID: infectious disease; NID: non-infectious disease; Pos, positive; Neg, negative; PPV, positive predictive value; NPV, negative predictive value. Mismatch: Positive results in tNGS do not match positive results in CMTs.

The sensitivity of tNGS was significantly higher than that of CMTs (81.25% vs 45.83%, *P* < 0.001). Additionally, 18 patients (34.62%) tested positive for both tNGS and CMTs, while 8 patients (15.38%) tested negative for both methods. Among the 18 cases with dual-positive results, 13 cases (72.22%) showed complete concordance, 4 cases (22.22%) showed partial concordance, and 1 case (5.56%) showed discordance (Fig. 5).

### Comparison of diagnostic performance between NTS and CMTs

CMTs failed to identify any effective pathogens in 121 out of 171 samples, whereas NTS detected 69 pathogens in CMTs-negative samples. On the contrary, among the pathogens not correctly identified by NTS, CMTs detected a total of four cases, including two cases of *Mycobacterium tuberculosis*, one case of Cryptococcus, and one case of Candida.

**Fig 6:**
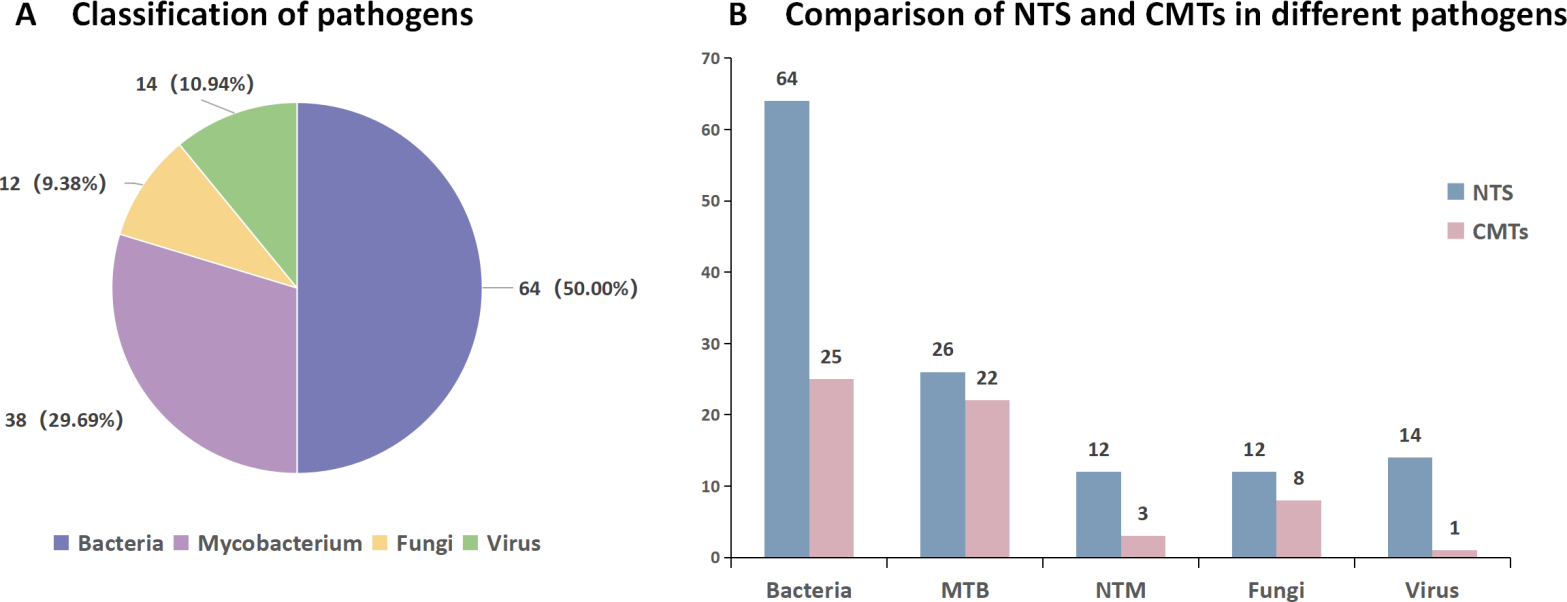
Subgroup analysis of pathogens detected by NTS. (**A**) The proportion of different pathogens in the identified pathogens. (**B**) Comparison of positive cases of NTS and CMTs for different pathogens in patients with identified pathogens.

NTS demonstrates superior detection capabilities across various categories of pathogens compared to CMTs, including bacteria (64 vs 25), MTB (26 vs 22), NTM (12 vs 3), fungi (12 vs 8), and viruses (14 vs 1). Meanwhile, NTS demonstrates significantly higher positivity rates than CMT (*P* < 0.05) for confirmed pathogens, including bacteria (96.97%), Mycobacterium (95.00%), fungi (75%), and viruses (100%) (Fig. 6). Additionally, NTS identified Legionella, *Chlamydia psittaci*, *Coxiella burnetii*, *Pneumocystis jirovecii*, Fusarium, coronaviruses, and rhinoviruses, none of which were detected by CMTs.

### Application of NTS in Mycobacterium complex

**Fig 7:**
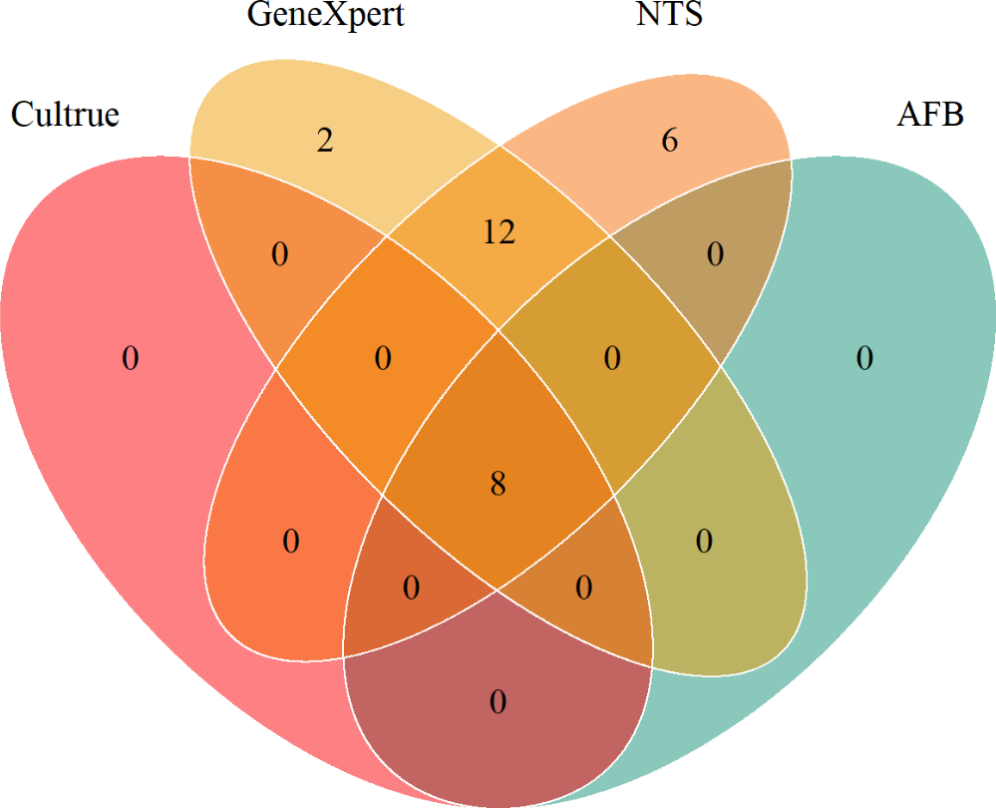
Venn diagram of positive tests for patients of pulmonary tuberculosis. GeneXpert: Xpert MTB/RIF; Culture*: Mycobacterium tuberculosis* culture; NTS: nanopore-targeted sequencing; AFB: acid-fast bacilli smear.

**Fig 8:**
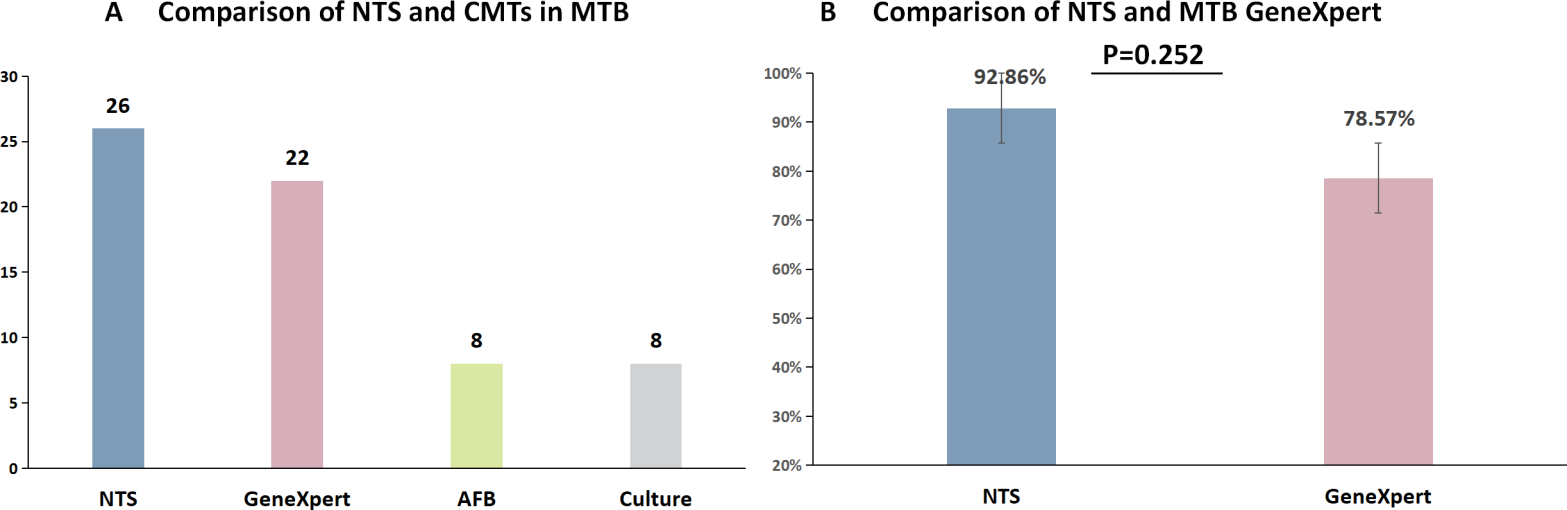
The diagnostic ability of NTS and CMTs in MTB. (**A**) The number of positive MTB cases was compared between NTS and different traditional diagnostic methods. (**B**) Comparison of Sensitivity between NTS and MTB GeneXpert.

NTS has exhibited notable advantages over traditional diagnostic methods for MTB, with a comparison among NTS (26 cases), MTB GeneXpert (22 cases), smear-positive cases (8 cases), and culture (8 cases) (Fig. 7). The sensitivity of NTS, in comparison to GeneXpert, stands at 92.86% versus 78.57% (*P* > 0.05, *P* = 0.252), showcasing a 14.29% increase in sensitivity. However, this improvement did not achieve statistical significance (Fig. 8). Additionally, NTS directly identified the specific NTM species in three smear-positive patients and successfully identified all NTM species in nine patients with negative results using CMTs (Table 1).

**TABLE 1:** All positive cases in non-tuberculous mycobacteria

No.	Accompanying disease	CMTs (acid-fast smear）	NTS
1	Bronchiectasis	Neg	*Mycobacterium intracellulare*
2	Diabetes	Pos	*Mycobacterium kansensis*
3	Bronchiectasis	Neg	*Mycobacterium abscessus*
4	None	Neg	*Mycobacterium avium* complex
5	None	Neg	*Mycobacterium intracellulare*
6	Leukemia	Pos	*Mycobacterium avium* complex
7	Bronchiectasis	Pos	*Mycobacterium avium* complex
8	None	Neg	*Mycobacterium avium* complex
9	None	Neg	*Mycobacterium avium* complex
10	Sjögren’s syndrome	Neg	*Mycobacterium avium* complex
11	None	Neg	*Mycobacterium avium* complex
12	None	Neg	*Mycobacterium avium* complex

### Comparison of tNGS/NTS and CMTs in culturable pathogens

**Fig 9:**
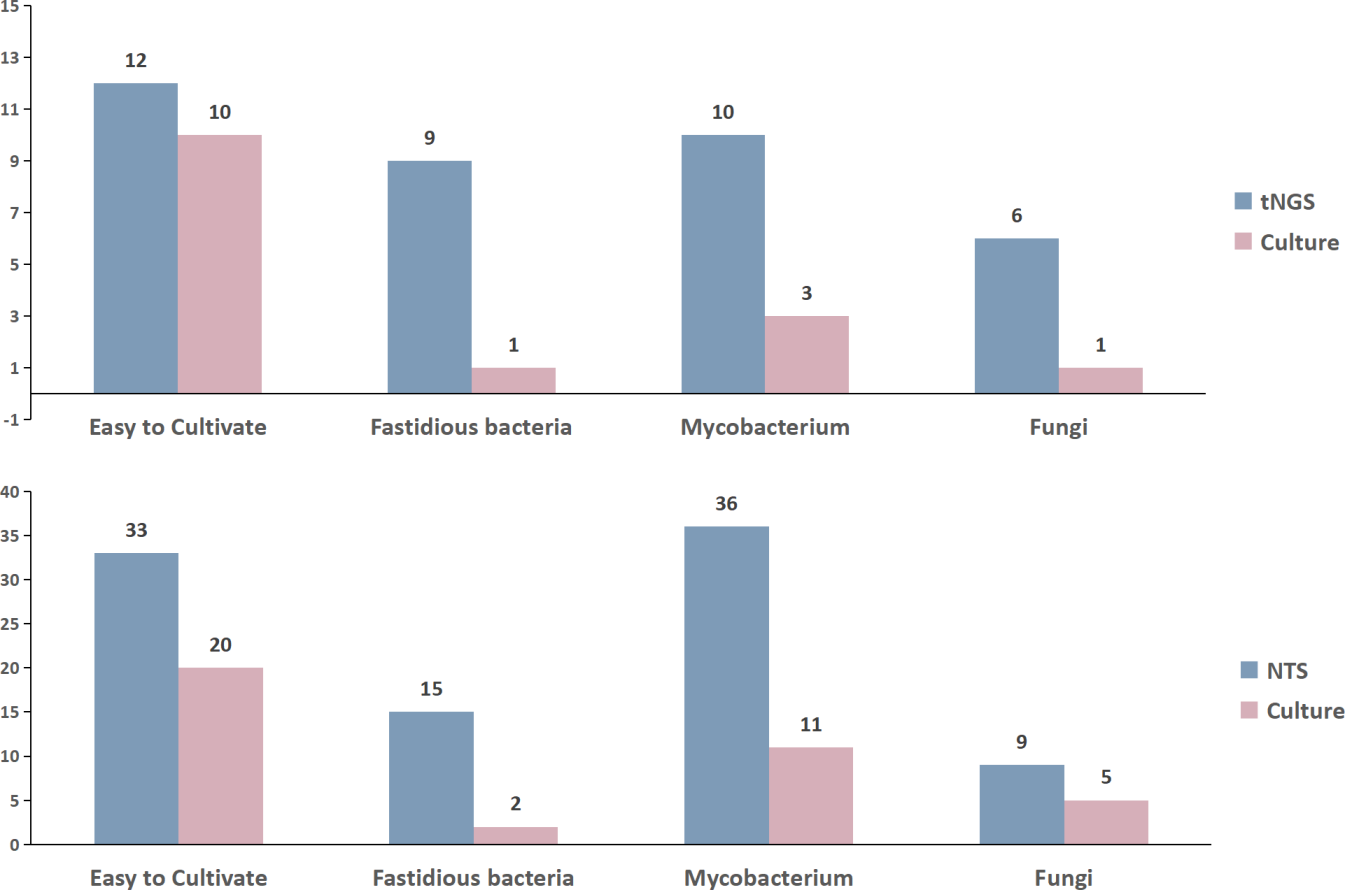
Comparison of tNGS/NTS and CMTs in culturable pathogens among those detected in this study.

Based on the pathogens detected by NTS, tNGS, and CMTs (Supplementary 4), cultivable pathogens were categorized into four groups: easily cultivable, fastidious bacteria, mycobacteria, and fungi. We then compared the quantities of pathogens detected by different detection methods. Initially, we compared tNGS and CMTs, revealing a pre-sampling antibiotic usage rate of 75% in easily cultivable bacteria, with detection counts of (12 vs 10), 77.78% in fastidious bacteria with counts of (9 vs 1), and 54.55% in branching bacteria with counts of (10 vs 3). The detection count comparison for fungi was (6 vs 1). Subsequently, similar comparisons between NTS and CMTs showed rates of 79.41%, 73.33%, and 50.00% in easily cultivable, fastidious, and branching bacteria, respectively, with respective detection counts of (33 vs 20), (15 vs 2), and (36 vs 11). The detection count comparison for fungi was (9 vs 5) (Fig. 9).

**Fig 10:**
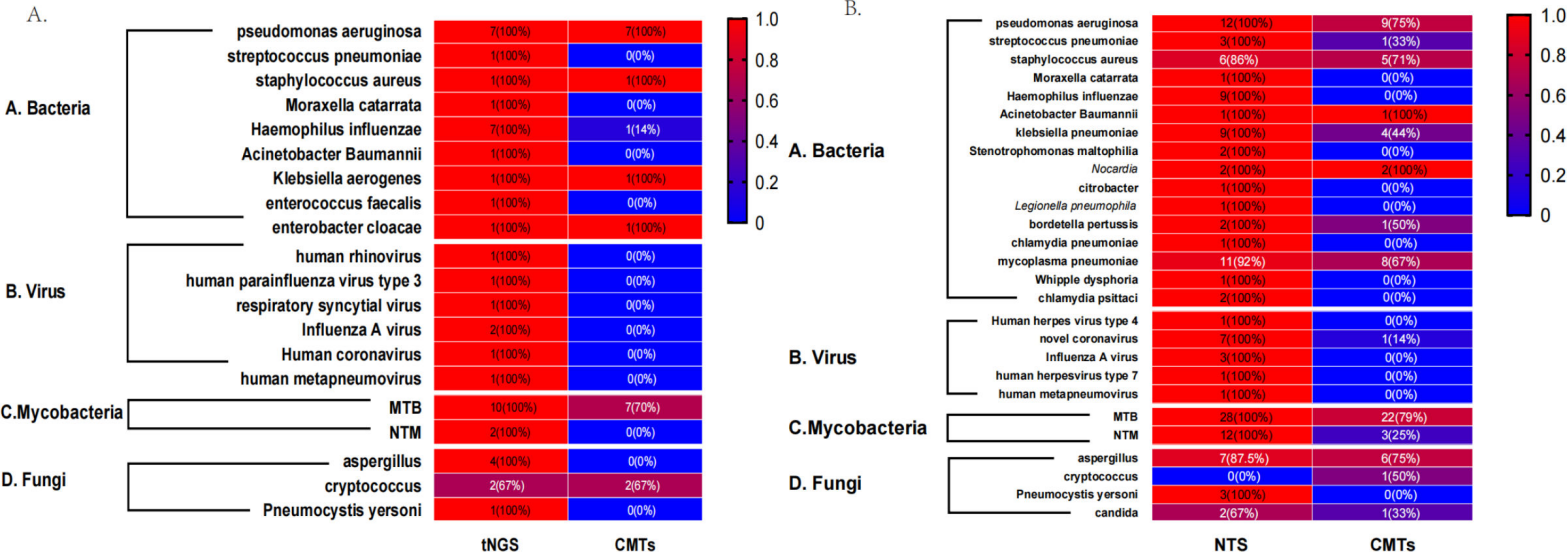
Heat map displaying the distribution of pathogens detected by (**A**) tNGS and CMTs (**B**) NTS and CMTs. The positive rate of each detection method was calculated using clinically confirmed pathogens as the gold standard.

Finally, using clinically confirmed pathogens as the gold standard, we calculated the positivity rates for each detection method and generated a heat map (Fig. 10).

## DISCUSSION

In this study, we separately assessed the overall sensitivity and specificity of tNGS and NTS compared to CMTs. We found that both targeted second-generation sequencing and nanopore-targeted sequencing technologies exhibited superior sensitivity. Additionally, we conducted a comparison of the overall sensitivity and specificity between tNGS and NTS for the first time, revealing on significant difference between the two. This finding provides robust support for their feasibility in clinical applications.

Currently, there are limited studies on the detection capabilities of tNGS. In a retrospective study involving 102 patients, the sensitivity of tNGS in BALF was 80.95% (22). In our study, the sensitivity of tNGS was 79.59%, showing a 46.91% increase compared to CMTs, consistent with the results of the aforementioned study. Additionally, there are several studies on the detection capabilities of NTS. In a study by Zhou et al. with 218 patients, NTS showed a sensitivity of 77.2%, a significant increase of 47.9% compared to CMTs (20). In a study by Fu et al. involving 436 patients, NTS detected pathogens in 88.7% of patients, while culture methods detected only 37.8% (23). Another study by Gu et al. with 146 patients reported that NTS detected pathogens in 91.1% of samples, while culture methods detected only 25.3% (24). In our study, we found that the sensitivity of NTS was 75.16%, representing a 42.48% increase compared to CMTs, consistent with the aforementioned studies.

For the pathogens culturable by CMTs, we further analyzed the number of positive cases identified by CMTs, which was significantly lower compared to tNGS and NTS. The significant difference in sensitivity between CMTs, tNGS, and NTS may be attributed to the coverage of pathogens by empirical antibiotics before routine cultivation, with tNGS and NTS being much less affected by antibiotics (25, 26). Moreover, nanopore sequencing has been previously used to identify antibiotic resistance genes (27). Additionally, some bacteria from the community in this study, such as *M. pneumoniae* and *C. psittaci*, are difficult to cultivate. Bacteria like *Streptococcus pneumoniae*, *Klebsiella pneumoniae*, and *Haemophilus influenzae* are fastidious bacteria, with strict requirements for culture environments and media (28, 29) . In our study, the antibiotic usage rate before patient sample cultivation was 78.5%. Under such a high proportion of empirical antibiotic treatment, the decreased sensitivity of CMTs seems inevitable. Furthermore, our study found that for pathogens like Legionella, *C. psittaci*, Rickettsia, *P. jirovecii*, and Aspergillus, which are challenging to cultivate, NTS demonstrated excellent detection capabilities. However, it is noteworthy that CMTs exhibited higher sensitivity in detecting Cryptococcus and other fungi, which was less sensitive for tNGS and NTS potentially due to their thick capsule (30).

Additionally, our study analyzed the detection capabilities of NTS in patients with suspected chronic pulmonary tuberculosis. The results revealed that NTS detected tuberculosis and concurrently identified NTM, some of which were co-infected with MTB. Given the clinical resemblance between MTB and NTM, the identification of NTM is a common finding in studies focusing on suspected tuberculosis cases (31–33). In this study, NTS exhibited a significant advantage in directly identifying NTM, especially when patients tested negative for AFB. Moreover, NTS demonstrated higher sensitivity compared to MTB GeneXpert testing for MTB, although the difference was not statistically significant, consistent with previous research findings (13). Considering the slow growth of *M. tuberculosis*, characterized by long culture cycles and times, NTS demonstrated a markedly shorter turnaround time of approximately 8–14 h (23). Additionally, the uncertainties arising from potential poor cultivation or microbial contamination in traditional cultures may impede rapid clinical diagnoses. These findings suggest that NTS and GeneXpert complement each other in diagnosing tuberculosis, and NTS exhibits robust capabilities in directly identifying NTM. Nano-pore sequencing is increasingly utilized in tuberculosis detection, and results from methods based on nanopore sequencing and Illumina platform whole-genome sequencing have shown 100% concordance in detecting drug-resistant mutations in *M. tuberculosis* (34). Since drug resistance in *M. tuberculosis* is primarily caused by point mutations at specific genetic targets, NTS offers significant promise for the rapid diagnosis of drug-resistant tuberculosis (35).

In this study, we not only assessed the overall diagnostic capabilities of tNGS and NTS but also conducted an initial comparison between these two distinct methodologies. Furthermore, we performed subgroup analyses from a clinical perspective to gain further insights into the clinical applications of NTS. However, the study has some limitations. First, it is a single-center retrospective study, and the populations for the tNGS and NTS comparison were not consistently matched. However, we made efforts to maximize the study sample size to mitigate potential biases in the comparison between NTS and CMTs. Second, due to the small size of the tNGS samples, no further subgroup analysis was conducted unfortunately. Third, the high pre-testing antibiotic usage rate may contribute to the decreased sensitivity of CMTs, potentially introducing bias to the results. However, our results generally align with findings from other studies. Notably, the experimental turnaround time and reporting time for NTS are significantly shorter than those for CMTs, mNGS, and other methods. Moreover, in China, the cost of NTS is approximately 1,000 RMB (about 150 USD), merely a quarter of the price of mNGS. Clearly, NTS has the potential to expedite and cost-effectively aid in the early diagnosis of respiratory infections.

### Conclusion

In BALF from patients with pulmonary infections, the ability to detect pathogens demonstrates that NTS and tNGS have significant overall advantages over conventional microbial testing (CMT) systems. Particularly in cases involving antibiotic use, they exhibit distinct advantages for fastidious bacteria and uncultivable pathogens. Additionally, NTS complements EXPERT in diagnosing *M. tuberculosis*.

## Data Availability

The sequence data are available in the NCBI SRA under NCBI BioProject ID PRJNA1092574.

## SUPPLEMENTAL MATERIAL

Supplemental material spectrum.00026-24-s0001.docx tNGS testing, NTS testing, CMTs testing, and culturable pathogens.

ASM does not own the copyrights to Supplemental Material that may be linked to, or accessed through, an article. The authors have granted ASM a non-exclusive, world-wide license to publish the Supplemental Material files. Please contact the corresponding author directly for reuse.
