# Supplementary material for: Clinical application of nanopore-targeted sequencing technology in bronchoalveolar lavage fluid from patients with pulmonary infections
Source: Microbiol Spectr. 2024 Apr 30;12(6):e00026-24. doi: 10.1128/spectrum.00026-24 (PMC11237526; doi:10.1128/spectrum.00026-24)
Supplement: Supplemental material — tNGS testing, NTS testing, CMTs testing, and culturable pathogens. [file spectrum.00026-24-s0001.docx]

***Supplementary 1，Detailed procedure of tNGS testing***

*tNGS testing: BALF samples (>5mL, placed in sterile sputum containers) are collected and immediately transported to a commercial laboratory (Hangzhou KingMed Diagnostics Laboratory (Hangzhou, China) using dry ice for testing.*

*Table 1. Instruments and equipment used*

| *Instrument name* | *Model* | *Manufacturer and Producer* |
| --- | --- | --- |
| *Gene sequencer* | *KM MiniSeqDx-CN* | *Kingcreate,Guangzhou* |
| *oscillating mixer* | *VORTEX-5* | */* |
| *low-speed centrifuge* | *JW-2002H* | */* |
| *PCR thermal cycler* | *Veriti Dx 96 Well Thermal Cycler* | *Thermo Fisher* |
| *Qubit quantifier* | *Qubit dsDNA HS Assay Kit* | *Invitrogen* |
| *high-speed centrifuge* | *eppendorf* | */* |
| *automated nucleic acid*  *and protein analysis system* | *Qsep100* | */* |
| *automated nucleic acid extractor* | *Auto-Pure96* | *[Allsheng,Hangzhou](https://www.allsheng.com.cn/product_cont_79.html)* |
| *48-channel purification system* | *NAP10* | *Kingcreate,Guangzhou* |
| *library preparation reagents* | *KS608-100HXD96* | *Kingcreate,Guangzhou* |
| *nucleic acid extraction or purification reagents* | *R6672B-F-48* | *[Magentec](http://www.magentec.com.cn/),Guangzhou* |
| *sequencing reagent kit* | *KS107-CXR* | *Kingcreate,Guangzhou* |

*Sample Preprocessing*

*Bronchoalveolar lavage fluid*

*Place the sampling tube on a vortex mixer and vortex thoroughly for 30 seconds; b) Transfer 1.3 mL of the sample to a new 1.5 mL centrifuge tube for pathogen enrichment by high-speed centrifugation at 12000 rpm for 5 minutes;*

*After centrifugation, remove the supernatant, retain 500 μL (manual extraction) or 250 μL (automated extraction) of the sample;*

*Take 500 μL (manual extraction) or 250 μL (automated extraction) of the sample to the bead-milling tube of the extraction reagent kit, add 50 μL (manual extraction) or 25 μL (automated extraction) of lysis buffer, tighten the lid, place it in a cell disruptor (4500-5000 rpm, shake for 30s, pause for 20s, repeat 2 times, totaling 3 cycles for 90s) or a mini-vortexer (set to maximum speed, shake for 10 minutes) for cell disruption;*

*After cell disruption, centrifuge at 12000 rpm for 5 minutes, take 500 μL (manual extraction) or 250 μL (automated extraction) of the supernatant for nucleic acid extraction; (viscous bronchoalveolar lavage fluid samples can be liquefied by adding 0.08 M DTT liquefaction reagent following the sputum sample processing method*

*2）Positive control*

*a) After thorough mixing, take 50 μL of the positive control and dilute it to 500 μL using RNase-free water;*

*b) Take 495 μL of the diluted positive control to the bead-milling tube of the extraction reagent kit, add 5 μL of exogenous internal reference and 50 μL of SDS, tighten the lid, place it in a cell disruptor (4700 rpm, shake for 45s, pause for 20s, repeat 2 times, totaling 3 cycles for 135s) for cell disruption;*

*c) After cell disruption, centrifuge at 12000 rpm for 5 minutes, take 400 μL (manual extraction) or 250 μL (automated extraction) of the supernatant for nucleic acid extraction;*

*3）Negative control*

*a) Take 495 μL of the negative control to the bead-milling tube of the extraction reagent kit, add 5 μL of exogenous internal reference and 50 μL of SDS, tighten the lid, place it in a cell disruptor (4700 rpm, shake for 45s, pause for 20s, repeat 2 times, totaling 3 cycles for 135s) for cell disruption;*

*b) After cell disruption, centrifuge at 12000 rpm for 5 minutes, take 400 μL (manual extraction) or 250 μL (automated extraction) of the supernatant for nucleic acid extraction.*

*2. Nucleic acid extraction*

*Nucleic Acid Extraction (Magnetic Bead Method): In a 2ml homogenization tube, add 20μl of Proteinase K; after vortex mixing, transfer 250μL of the sample to the homogenization tube, invert to mix, and incubate at 55 degrees Celsius for 10 minutes. Remove the sample, and incubate at 55-60 degrees Celsius for another 10 minutes. Centrifuge at 10,000xg for 3 minutes. Start the fully automated extractor, place the sample and reagents accordingly. After approximately 45 minutes, finish the extraction, remove the 96-well plate, transfer the product to clean 8-tube strips, and store at -20 to 8°C. Measure the nucleic acid concentration of the samples using Qubit 4.0, follow the instructions of the Qubit dsDNA HS Assay Kit (Invitrogen Corporation), and record the nucleic acid concentration.*

*3. Library construction*

*3.1cDNA Synthesis*

*1)Thaw the first-strand synthesis buffer and cDNA synthesis primers on ice in advance, briefly vortex for 5 seconds, pulse-centrifuge for 3 seconds, and keep them on ice for later use.*

*2）Take out the first-strand synthesis enzyme before use, gently flick to mix, pulse-centrifuge for 3 seconds, and keep it on ice for later use.*

*3）Based on the nucleic acid concentration, adjust the sample volume accordingly. If the concentration is greater than or equal to 10 ng/μL, add 100 ng volume to make up to 14 μL and put it into a pre-labeled 96-well PCR plate. If the nucleic acid concentration is too low or less than 10 ng/μL, add 14 μL to the labeled 96-well PCR plate. Use 14 μL of extracted pure water quality control NTC1 and add it to the labeled 96-well PCR plate. Also, add 14 μL of nuclease-free water to the labeled 96-well PCR plate as the library construction pure water quality control NTC2 for library quality control. Add 2 μL each of the positive and negative controls to the labeled 96-well PCR plate and make up to 12 μL. Then, add 2 μL of cDNA synthesis primers to each well, vortex mix for 10 seconds, pulse-centrifuge for 3 seconds, and place in the PCR instrument for incubation at 65°C for 2 minutes, followed by holding at 4°C.*

*4）Perform the following steps with the reaction tubes on ice: sequentially add 2 μL of first-strand synthesis buffer and 2 μL of first-strand synthesis enzyme, gently vortex mix for 10 seconds, pulse-centrifuge for 3 seconds, and place in the PCR instrument. Run the program for one-strand cDNA synthesis. After the program ends, remove the reaction plate from the PCR instrument, pulse-centrifuge for 3 seconds, and place it on ice. The cDNA synthesis program is as shown in Table 4:*

*Table4 cDNA Synthesis Program*

| *Temperature* | *Time* | *Cycle Number* |
| --- | --- | --- |
| *25℃* | *5 minutes* | *1* |
| *37℃* | *45 minutes* | *1* |
| *85℃* | *5 seconds* | *1* |
| *4℃* | *∞* | *1* |

*3.2 Target Region Enrichment*

*2.1 Configuration of the Target Region Enrichment Reaction System*

*1）Thaw and mix the multiplex PCR premix, vortex, centrifuge, and place on ice;*

*2）Add 9.5 μL of cDNA product to the mixture；*

*3）Seal the membrane, mix by shaking, centrifuge, place in the PCR instrument, and run the following program for target region enrichment.*

*2.2 Setting of Target Region Enrichment PCR Program*

| *Temperature* | *Time* | *Cycle Number* |
| --- | --- | --- |
| *95℃* | *3 minutes* | *1* |
| *95℃* | *30 seconds* | *28* |
| *60℃* | *30 seconds* |  |
| *72℃* | *30 seconds* |  |
| *72℃* | *1 minute* | *1* |
| *4℃* | *∞* | *1* |

*2.3 Purification of Target Region Enrichment PCR Products*

*1）Before purification, equilibrate the purification magnetic beads to room temperature and prepare 80% ethanol；*

*2）Briefly centrifuge the PCR product, add 25 μL of purification magnetic beads, mix by shaking, and incubate for 5 minutes；*

*3）Briefly centrifuge, place on a magnetic stand, remove the supernatant after clarification, add 100 μL of 80% ethanol, let it stand for 30 seconds, then remove the supernatant；*

*4）Repeat step 3) once；*

*5）Briefly centrifuge, use a 10 μL pipette tip to remove the remaining residual ethanol, air dry the magnetic beads at room temperature for about 3-5 minutes, and observe that the surface of the magnetic beads does not reflect light.*

*6）Add 13.5 μL of nuclease-free water, mix and incubate for 5 minutes；*

*7）Briefly centrifuge, place on a magnetic stand, transfer 11.5 μL of the product to the library amplification premix；*

*3 Library Amplification*

*3.1 Configuration of Library Amplification Reaction System*

*1）Thaw and mix the library amplification premix, vortex, centrifuge, and place on ice；*

*2）Add 11.5 μL of purified and enriched products to the mixture；*

*3）Seal the membrane, mix by shaking, centrifuge, place in the PCR instrument, and run the following program for library amplification。*

*3.2 Setting of Library Amplification PCR Program*

| *Temperature* | *Time* | *Cycle Number* |
| --- | --- | --- |
| *95℃* | *3 minutes* | *1* |
| *95℃* | *30 seconds* | *10* |
| *60℃* | *30 seconds* |  |
| *72℃* | *30 seconds* |  |
| *72℃* | *1 minute* | *1* |
| *4℃* | *∞* | *1* |

*3.3 Library Purification*

*1）Before purification, equilibrate the purification magnetic beads to room temperature and prepare 80% ethanol.*

*2）Take out the PCR product for brief centrifugation, add 25 μL of purification magnetic beads, shake well to mix, and incubate for 5 minutes.*

*3）Briefly centrifuge, place on a magnetic stand, remove the supernatant after clarification, add 100 μL of 80% ethanol, let it stand for 30 seconds, then remove the supernatant.*

*4）Repeat step 3) once.*

*5）Briefly centrifuge, use a 10 μL pipette tip to remove the remaining residual ethanol, air dry the magnetic beads at room temperature for about 3-5 minutes, and observe that the surface of the magnetic beads does not reflect light.*

*6）Add 22 μL of nuclease-free water, mix and incubate for 5 minutes.*

*7）Briefly centrifuge, place on a magnetic stand, transfer 20 μL of the product to a pre-labeled 96-well plate for quantification.*

*3.4 Library Quality Control*

*1）Measure the concentration of the sample library using Qubit 3.0/4.0 according to the instructions of the Qubit dsDNA HS Assay Kit (Invitrogen), and record the library concentration. If the library concentration is <0.5 ng/μL, it indicates a library construction failure and requires re-construction.*

*4 Library Pooling and Quality Control*

*4.1 Library Pooling*

*Each library is pooled in equal quantities (within the range of 20 ng to 50 ng based on the library concentration), and the library sampling volume is calculated using the following formula:*

*Library Sampling Volume (ng) / Library Concentration (ng/μL) = Sampling Volume (μL)*

*4.2 Pooling Library Quality Control*

*1）Measure the concentration of the pooled library using Qubit 3.0/4.0 according to the instructions of the Qubit dsDNA HS Assay Kit (Invitrogen), and record the library concentration.*

*2）Library size quality control: Use an automated nucleic acid and protein analyzer (Qsep100) to detect the library fragment size. Use the Standard Cartridge Kit (S2) reagent kit for detection, following the instructions provided with the kit. The detected library fragment size should be around 250-350 bp as shown in Figure 2.*

**Note: If there is no peak or the proportion is less than 20% in the 250-350 bp range, it indicates a library construction failure or poor quality, requiring library reconstruction. If there are large fragments present, proceed with step 4.3 and then repeat the library quality control steps in 4.2.*

*4.3 Library Pooling Purification*

*1）Before purification, equilibrate the purification magnetic beads to room temperature and prepare 80% ethanol.*

*2）Take out 50 μL of the pooled library (if the volume of the pooled library is less than 50 μL, supplement with nuclease-free water to reach 50 μL), add 35 μL of purification magnetic beads, shake well, and incubate for 5 minutes.*

*3）Instantly centrifuge, place on a magnetic stand, transfer all the supernatant to a new tube containing 40 μL of purification magnetic beads, shake well, and incubate for 5 minutes.*

*4）Instantly centrifuge, place on a magnetic stand, remove the supernatant, add 100 μL of 80% ethanol, let it stand for 30 seconds, then remove the supernatant.*

*5）Repeat step 4) once.*

*6）Instantly centrifuge, use a 10 μL pipette tip to remove the remaining residual ethanol, air dry the magnetic beads at room temperature for about 3-5 minutes, and observe that the surface of the magnetic beads is non-reflective. 7）Add 22 μL of nuclease-free water, mix well, and incubate for 5 minutes.*

*8）Instantly centrifuge, place on a magnetic stand, and draw up 20 μL of the supernatant into a new 0.2 mL PCR tube.*

*9）Re-perform library pooling quality control.*

*3.Sequencing*

*Utilizing the sequencing reaction universal reagent kit from Guangzhou Kingcreate Biotechnology Co., Ltd.*

*1. Preparation of Sequencing Consumables*

*1.1 Preparation of Reagent Tray*

*1.1.1 Remove the reagent tray and place it in a water bath at room temperature (19-25°C) to thaw for approximately 90 minutes. Do not submerge the entire tray, and ensure that the water level does not exceed the tray. If multiple trays are placed in the same water bath for thawing, extend the thawing time accordingly.*

*1.1.2 Check the bottom of the tray to ensure complete thawing of the reagents without ice crystals. Wipe the bottom dry, shake the tray to mix the reagents, and gently tap the tray on the countertop to reduce any air bubbles at the bottom.*

*1.2 Preparation of Sequencing Chips*

*1.2.1 Retrieve a new sequencing chip from 2-8°C and allow the unopened chip to stand at room temperature for 30 minutes to avoid repeated refrigeration.*

*1.2.2 Put on a new pair of powder-free gloves and remove the sequencing chip from the foil packaging. If there is dust on the sequencing chip, carefully wipe the glass surface with a lint-free paper, avoiding wiping the black spacer.*

*Note: Before use, check whether the chip is intact and undamaged, and if there are any abnormal phenomena such as the black spacer being raised. If any anomalies are found, contact an engineer for assistance.*

*2. Preparation of Sequencing Library*

*2.1 Before adding the library to the reagent tray, dilute and denature it. Convert the library concentration using the formula: Library molar concentration (nmol/L) = Library quantification concentration (ng/μL) × 1000000/660/average library fragment size (bp). Then, according to the formula, dilute the library in a 1.5 mL low-bind centrifuge tube (diluting each library concentration to 1 nmol/L), where the volume of nuclease-free water added to the diluted library (in μL) = Library molar concentration (nmol/L) × volume of library added for dilution (μL) / target concentration of the diluted library (1 nmol/L) - volume of library added for dilution (μL). Before mixing, based on the ratio of the theoretical sequencing throughput, mix the diluted libraries in proportion to ensure a sequencing throughput: mixed volume = 1:1. It is recommended that the theoretical data volume be less than the sequencing throughput, which determines the number of mixed libraries. Combine the sequencing libraries into one tube to obtain the original mixed library (concentration of 1 nmol/L) and place it in a new 1.5 mL low-bind centrifuge tube.*

*2.2 Place the sequencing buffer at room temperature 30 minutes in advance and then store it in a refrigerator at 4°C after thawing.*

*2.3 Prepare 0.1 mol/L sodium hydroxide diluent: Add 100 μL of 1 mol/L sodium hydroxide solution to a 1.5 mL low-bind centrifuge tube containing 900 μL of nuclease-free water, shake well, and briefly centrifuge for later use (prepare and use immediately, and use within 12 hours after preparation).*

*2.4 Add 5 μL of 1 nmol/L original mixed library and 5 μL of 0.1 mol/L sodium hydroxide diluent to a new 1.5 mL low-bind centrifuge tube, shake well, briefly centrifuge, and let it stand at room temperature for 5 minutes for denaturation, obtaining 10 μL of 0.5 nmol/L (500 pmol/L) diluted mixed library.*

*2.5 Pipette 990 μL of the mixed sequencing buffer into the above 10 μL tube of diluted mixed library to dilute it to 5 pmol/L, labeled as diluted mixed library C1.*

*2.6 Calculate the volume of diluted mixed library to be added according to the formula: Volume V1 (μL) of diluted mixed library = Target concentration C2 of sequencing mixed library (1 pmol/L) × Required volume V2 of sequencing mixed library (600 μL) / Concentration C1 of diluted mixed library (5 pmol/L).*

*2.7 Mix (V1) μL of 5 pmol/L diluted mixed library C1 and (600-V1) μL of sequencing buffer, shake well, briefly centrifuge, to obtain 600 μL of 1 pmol/L sequencing mixed library.*

*2.8 Use a 1 mL pipette tip to pierce hole 16 of the foil, and take 500 μL of 1 pmol/L sequencing mixed library.*

*2.9 Create a Sequencing Program*

*2.10 Run the Sequencing Program*

*4. Bioinformatics Analysis*

*Bioinformatics analysis was conducted using the independently developed "Pathogenic Microorganism Data Analysis Management System" v3.3.4 from Guangzhou Jinqi Rui Biotechnology Co., Ltd. The data quality requirements were as follows: Q30 ≥ 75%, minimum number of original reads ≥ 50k, and internal reference gene amplification reads ≥ 200.*

*The specific analysis workflow is as follows:*

*a) The raw sequencing data obtained from the sequencer was split to obtain sequencing data in fastq format using the bcl-convert software (version 00.000.000.3.10.5).*

*b) Pre-alignment processing of the split sequence data was performed using fastp (version 0.20.1) to filter out adapter sequences, low-quality sequences, sequences with excessive Ns, and sequences that are too short after filtering, resulting in usable sequences for further analysis.*

*c) The alignment software Bowtie2 (version 2.4.1) was used to align the cleaned effective sequences with the tNGS in-house database using the --very-sensitive alignment parameters.*

*The tNGS in-house database collected and organized commonly used public databases such as NCBI, GenBank, refseq, DDBJ, and authoritative literature-recommended genome sequences. It filtered out artificial sequences like plasmids and phages, and evaluated, cleaned, and annotated database sequences based on assembly level, submission source, sequencing method, clustering analysis, etc. Pathogen resolution information was compiled based on product scope, literature research, clinical reports, etc.*

*All processes at KingMed Diagnostics Laboratory referred to the following literature(1, 2(1, 2)*

1. Gaston DC, Miller HB, Fissel JA, Jacobs E, Gough E, Wu J, Klein EY, Carroll KC, Simner PJ. 2022. Evaluation of Metagenomic and Targeted Next-Generation Sequencing Workflows for Detection of Respiratory Pathogens from Bronchoalveolar Lavage Fluid Specimens. J Clin Microbiol 60:e0052622.

2. Li S, Tong J, Liu Y, Shen W, Hu P. 2022. Targeted next generation sequencing is comparable with metagenomic next generation sequencing in adults with pneumonia for pathogenic microorganism detection. J Infect 85:e127-e129.

***Simplified Version of SOP***

*Using a highly multiplex PCR combined with NGS technology, specific primers were designed to target highly conserved regions of 198 respiratory pathogens. PCR amplification and enrichment of target pathogens were performed in a single amplification tube, followed by the connection of sequencing adapters to distinguish sample sources in the second round of PCR. Targeted next-generation sequencing (tNGS) was conducted using a gene sequencer to obtain sequencing data. Bioinformatics software was then used to filter the sequencing data, align it with reference genomes, and interpret the pathogen detection results, achieving broad-spectrum and precise pathogen detection.*

*(1) Respiratory 198 items (pharyngeal swab, sputum, bronchoalveolar lavage fluid samples):*

*Main equipment:*

*①Auto-Pure 96 fully automated nucleic acid extractor; ②Veriti Dx 96-Well Thermal Cycler PCR Amplifier; ③Qubit 4.0 Fluorometer; ④KM MiniseqDx-CN gene sequencer*

*Reagents:*

*①Extraction reagent: R6672B-F-48 nucleic acid extraction or purification reagent (Guangzhou [Magentec](http://www.magentec.com.cn/) Biotechnology Co., Ltd.)*

*②Library construction reagent: KS608-100HXD96 respiratory pathogen microbial multiplex detection test kit (Guangzhou Kingcreate Biotechnology Co., Ltd.)*

*③Sequencing reagent: KS107-CXR sequencing reaction universal reagent kit (Guangzhou Kingcreate Biotechnology Co., Ltd.)*

*Methods:*

*①Sample pretreatment:*

*Pharyngeal swab samples: Place the sampling tube on a vortex mixer and vortex thoroughly for 30 seconds to wash off viruses and virus-containing cells adhering to the swab. Transfer 500μL (manual extraction) or 250μL (automated extraction) of the sample to the extraction reagent vial, and add 50μl (manual extraction) or 25μl (automated extraction) of lysis solution. Tighten the cap and place it in a bead beater (4500-5000rpm, shake for 30s, pause for 20s, repeat 2 times, shake for a total of 90s) or a mini oscillator (set to maximum speed, shake for 10min) for disruption treatment. After disruption treatment, centrifuge at 12000rpm for 20s and take 500μL (manual extraction) or 250μL (automated extraction) of the supernatant for nucleic acid extraction.*

*Sputum samples: Add an equal volume of 0.08 M DTT liquefying reagent to the sample tube, mix by shaking, and let stand for 3-5 minutes until the sample is liquefied. Prepare a new 1.5mL centrifuge tube, aspirate 1.3mL of the liquefied sample, and centrifuge at high speed to enrich the pathogens, at a speed of 12000rpm for 5 minutes. After centrifugation, remove the supernatant, retaining 500μL (manual extraction) or 250μL (automated extraction) of the sample. Take 500μL (manual extraction) or 250μL (automated extraction) of the sample to the extraction reagent vial, and add 50μl (manual extraction) or 25μl (automated extraction) of lysis solution. Tighten the cap and place it in a bead beater (4500-5000rpm, shake for 30s, pause for 20s, repeat 2 times, shake 3 times in total for 90s) or a mini oscillator (set to maximum speed, shake for 10min) for disruption treatment. After disruption treatment, centrifuge at 12000rpm for 20s, take 500μL (manual extraction) or 250μL (automated extraction) of the supernatant for nucleic acid extraction.*

*Bronchoalveolar lavage fluid samples: Place the sampling tube on a vortex mixer and vortex thoroughly for 30 seconds. Aspirate 1.3mL of the sample into a new 1.5mL centrifuge tube for high-speed centrifugation to enrich the pathogens, at a speed of 12000rpm for 5 minutes. After centrifugation, remove the supernatant, retaining 500μL (manual extraction) or 250μL (automated extraction) of the sample. Take 500μL (manual extraction) or 250μL (automated extraction) of the sample to the extraction reagent vial, and add 50μl (manual extraction) or 25μl (automated extraction) of lysis solution. Tighten the cap and place it in a bead beater (4500-5000rpm, shake for 30s, pause for 20s, repeat 2 times, shake 3 times in total for 90s) or a mini oscillator (set to maximum speed, shake for 10min) for disruption treatment. After disruption treatment, centrifuge at 12000rpm for 20s, take 500μL (manual extraction) or 250μL (automated extraction) of the supernatant for nucleic acid extraction; (Thick bronchoalveolar lavage fluid samples can be processed in the same way as sputum samples, adding 0.08 M DTT liquefying reagent for sample liquefaction).*

*②Nucleic acid extraction (magnetic bead method): In a 2ml homogenization tube, add 20ul Proteinase K; after mixing, transfer 250μL of the sample to the homogenization tube, invert and mix, and incubate at 55 degrees for 10 minutes. Remove the sample, and incubate at 55-60 degrees for 10 minutes. Centrifuge at 10,000xg for 3 minutes. Start the fully automatic extractor, place the corresponding sample and reagent. After about 45 minutes, remove the 96-well plate, transfer the product to a clean 8-tube, and store at -20~8°C. Use Qubit 4.0 to determine the nucleic acid concentration of the sample, following the instructions of the Qubit dsDNA HS Assay Kit (Invitrogen company), and record the nucleic acid concentration.*

*③Library construction: Synthesize cDNA, target region enrichment, library amplification, library purification, use Qubit 4.0 to determine the library concentration and record the library concentration. Pool each library at equal quantities (within the range of 20ng~50ng based on the concentration at the time of release), and calculate the library sampling volume according to the following formula: Library sampling amount (ng) / Library concentration (ng/μL) = Sampling volume (μL). Perform library quality control: Use Qubit 4.0 to determine the concentration of the pooled library and record the library concentration; use an automated nucleic acid protein analyzer (Qsep100) to detect the size of the library fragments, following the instructions of the reagent box, and ensure that the library fragment size is around 250-350 bp.*

*④Sequencing: Prepare reagent tubes and chips, prepare the sequencing library, and start sequencing.*

*⑤Bioinformatics analysis: Use the independently developed "Pathogen Microorganism Data Analysis Management System" v3.3.4 from Guangzhou Kingcreate Biotechnology Co., Ltd. for bioinformatics analysis.*

***Supplementary 1***

*Table 1. 198 respiratory pathogens were included in the panel of this study*

| *Bacterium (n=80)* | *Gram-positive*  *bacteria* | *Rhodococcus equi; Staphylococcus aureus; Streptococcus agalactiae; Streptococcus angina group; Streptococcus intermedius; Streptococcus pneumoniae; Streptococcus pyogenes; Micromonas; Corynebacterium diphtheriae; Mycobacterium asiatica; Mycobacterium avium; Mycobacterium avium complex group; Cryptic Mycobacterium; Mycobacterium Gordonii; Mycobacterium intracellular; Mycobacterium kansensis; Mycobacterium Malmo; Non-tuberculous mycobacterium; Mycobacterium scrofula; Mycobacterium Schweierii; Mycobacterium pithecus; Mycobacterium surga; Mycobacterium tuberculosis complex group; Mycobacterium toad; Mycobacterium abscessus; Mycobacterium corneum-abscess congregates; Mycobacterium chelonis; Sporadic Mycobacterium; Mycobacterium smegmatis; Nocardia; Nocardia abscessus; Nocardia africana; Nocardia stellata; Nocardia brasiliensis; Nocardia indented; Nocardia SAN Georges (Nocardia gelsenkirchen); Nocardia glandis; Nocardia otitis of guinea pig; Nocardia terpene; Arcanobacterium pyogenes; Whipple dysphoria; New Nocardia* |
| --- | --- | --- |
|  | *Gram-negative bacteria* | *Moraxella catarrata; Serophilus meningitidis; Acinetobacter baumannii; Acinetobacter joni; Acinetobacter ursinensis; Bacteroides fragilis; Holmbaute; Bordetella parapertussis; Bordetella pertussis; Brucella; Burkholderia cepacia; Burkholderia cepacia; Burkholderia cepacia complex group; Burkholderia malleens; Burkholderia melioides; Contaminated Burkholderia; Burkholderia polyphagia; Chrysoelisabeth anopheles; Escherichia pyosus meningosa; Enterobacter cloacae complex group; E. coli; Fusobacterium necrotica; Fusobacterium nucleatum; Haemophilus influenzae; Klebsiella aerogenes; Acid-producing Klebsik; Klebsiella pneumoniae; Klebsiella mutans; Legionella; Legionella bozeman; Legionella pneumophila; Legionella longicarensis; Legionella miktide; Pasteurella multocida; Proteus mirabilis; Pseudomonas aeruginosa; Serratia clay; Stenotrophomonas maltophilia* |
| *Fungi（n=32）* | | *Candida glabrata; Aflatus complex group; Aspergillus fumigatus; Aspergillus Niger complex group; Aspergillus terreus complex group; Candida albicans; Candida palmiformis; Candida tropicalis; Cryptococcus gerdt; Cryptococcus neoformans; Fusarium; Histopleura leymenae; Metaphylla; Crossstalk umbelliferous; Transverse pedunculus ramosus; Candida tiyemonda (Pichia tiyemonda); Mucor irregularis; Mucor racemose; Pichia azweiensis Kudri (Candida klurensis); Pneumocystis yersoni; Phyllotrichum; Rhizopus minutus; Rhizopus; Rhizopus delsoni; Rhizopus microsporus; Oryza L.; Sydospora; Sedospora cuspis; Saydospora poieri (Saydospora boydesi); Bacillus marneffei; Trichospora asasiensis* |
| *Others*  *(n=7)* | *Mycoplasmas* | *Mycoplasma pneumoniae; Ureaplasma parvos; Ureaplasma urealyticum* |
|  | *Chlamydia* | *Chlamydia pneumoniae; Chlamydia psittaci; Chlamydia trachomatis* |
|  | *Rickettsiae* | *Coxiella burnetii* |
| *Viruses (n=79)* | *DNA viruses* | *BK polyomavirus (human polyomavirus type 1); Human adenovirus type 1; Human adenovirus type 2; Human adenovirus type 21; Human adenovirus type 5; Human adenovirus type 55; Human adenovirus type 57; Human adenovirus type 6; Human adenovirus type 7; Human adenovirus type 3; Human adenovirus type 4; Human herpesvirus type 1 (HSV1); Human herpesvirus type 2 (HSV2); Human herpes virus type 3 (VZV); Human herpes virus type 5 (CMV); Human herpesvirus type 6; Human herpesvirus type 7; Human Boca virus type 1; Human Boca virus type 2; Human Boca virus type 3; Human Boca virus type 4; Human herpes virus type 4 (EBV); Human adenovirus; Human adenovirus group B; Human adenovirus group C; Human adenovirus group D; Human parvovirus B19; Human adenovirus type 11; Human adenovirus type 14; Human adenopathy wine type 34; Human adenovirus type 35; Human herpesvirus type 6A; Human herpesvirus 6B; JC polyomavirus (human polyomavirus type 2); WU Polyomavirus (human Polyomavirus type 4)* |
|  | *RNA viruses* | *Coxsackie virus A10; Coxsackie virus A16; Coxsackie virus A2; Coxsackie virus A5; Coxsackie virus A6; Coxsackie virus B3; Echovirus E18; Echovirus E30; enterovirus*  *; Enterovirus group A; Enterovirus A71 plastic; Enterovirus group B; Enterovirus Group C; Enterovirus group D; Enterovirus D68; Human coronavirus 229E; Human coronavirus HKU1; Human coronavirus NL63; Human coronavirus OC43; Human metapneumovirus; Human mumps virus type 2 (human parainfluenza virus type 2); Human mumps virus type 4 (human parainfluenza virus type 4); Human respiratory syncytial virus type A; Human respiratory syncytial virus B; Human respiratory virus type 1 (human parainfluenza virus type 1); Human respiratory virus type 3 (human parainfluenza virus type 3); Influenza A virus; Influenza A virus HIN1; Influenza A virus H3N2; Influenza A attacks H5N1; Influenza A virus H7N9; Influenza B virus; Influenza C virus; Influenza A virus H1N1 (2009); Influenza B virus Victora strain; Influenza B virus Yamagata strain; Measles virus; Mumps virus; Rhinovirus; Rhinovirus type A; Rhinovirus B type; Rhinovirus type C; Rubella virus; novel coronavirus.* |

***Supplementary 2，Detailed procedure of NTS testing***

*NTS testing: BALF samples (>5mL, placed in sterile sputum containers) are collected and immediately transported to a commercial laboratory (Hangzhou Dean Medical Laboratory (Hangzhou, China) using dry ice for testing.*

*Table 1. Instruments and equipment used*

| *Instrument name* | *Model* | *Manufacturer and Producer* |
| --- | --- | --- |
| *Desktop refrigerated centrifuge* | *BY-R320* | *Baiyang, Beijing* |
| *Vortex Mixers* | *QL-901* | *Qilinbeire, Haimen* |
| *High speed centrifuge* | *BY-G1200* | *Baiyang, Beijing* |
| *Tissue grinding homogenizer* | *TL2020S* | *Dinghaoyuan, Tianjing* |
| *Fluorescence quantizer* | *qubit 4.0* | *Thermo Fisher* |
| *Constant temperature water bath* | *DKT-200* | *Miou, Hangzhou* |
| *PCR instrument* | *Hema9600* | *Heima, Zhuhai* |
| *Nanopore sequencer* | *GridION MK1* | *Oxford Nanoporous Technology* |

*1. Sample Preprocessing*

*Liquefaction of alveolar lavage fluid (only for viscous samples), add a certain amount of liquefied fluid and 20% by volume 1:1 μ L XC, shake and mix well, shake at 1000 rpm on a constant temperature mixer for 1-3 minutes, incubate at 37 ℃ for 10 minutes (if the liquefaction is still viscous, increase the amount of liquefaction liquid appropriately for secondary liquefaction), take 1mL (the maximum volume of less than 1mL) and add it to a 1.5mL sterile centrifuge tube, centrifuge at 12000 rpm for 2 minutes, and discard 950 μ L supernatant (residual liquid approximately 30-40 μ L) . Add 200 μ L cracking solution LS, shake or blow with a gun head to suspend and disperse the precipitate, and react at 37 ℃ for 10 minutes. Add 100mg of glass beads and shake the wall breaker at 2000rpm for 8 minutes (pausing for 30 seconds every 1 minute) at high speed.*

*2. Nucleic acid extraction*

*Add 400 μ L cracking liquid LE, 20 μ L Proteinase K, blow and mix well, and absorb about 600 after brief centrifugation μ Add L supernatant to the prepared 96-well deep well plate in the 1st/7th row of holes, and place the 96-well plate and magnetic sleeve into the Hema nucleic acid automatic extractor according to the requirements of the instrument manual, and automatically run according to the set program. After the program is completed, transfer the nucleic acid solution from the 6/12 well to a 1.5 mL EP tube and measure the DNA concentration using a Qubit 3.0 fluorescence quantitative analyzer.*

*3. Library construction*

*3.1 Targeted amplification*

*Prepare a reverse transcription reaction mixture in a 0.2 mL PCR centrifuge tube according to the ratio in Table 2 (the reaction solution needs to be prepared and packaged on an ice box, and the ice box needs to be kept at low temperature), mix it gently, centrifuge briefly, and place it in a PCR machine for reverse transcription reaction. The reaction conditions are as follows: 37 ℃ for 15 minutes, 85 ℃ for 5 seconds, 4 ℃ hold。*

*Table 2. Reverse transcription reaction mixture*

| *Component* | *Per sample/volume（μL)* |
| --- | --- |
| *RNA* | *8* |
| *Reverse transcription reaction fluid* | *2* |
| *Total* | *10* |

*Prepare the required amount of PCR reaction mixture in a new centrifuge tube as shown in Table 3, and perform the PCR reaction according to the reaction conditions shown in Table 4 after completion.*

*Table 3. PCR Reaction System*

| *Component* | *Per sample/volume（μL)* |
| --- | --- |
| *Reverse transcript product* | *4μl* |
| *PCR amplification reaction solution* | *36μl* |
| *Total* | *40 μl* |

*Table 4 PCR Reaction Procedure*

| *Temperature* | *Time* | *Cycle* |
| --- | --- | --- |
| *95℃* | *2 min* | *1* |
| *95℃* | *15 s* | *35* |
| *60℃* | *2min 15 s* |  |
| *72℃* | *5 min* | *1* |
| *4℃* | *Hold* | *1* |

*After the reaction is completed, add 1.5X volume of magnetic beads for purification, blow and mix well, and let stand for 5 minutes; Transfer to a magnetic rack, wait for the solution to clear (about 2 minutes), discard the supernatant, and use 150 μ Wash twice with 70% -80% ethanol (do not disturb the magnetic beads), immediately remove and place the remaining ethanol on a magnetic rack with 10 μ L Pipette absorbs the solution, leaves it to dry for 2min, and finally washes it with 25 µ L ddH2O water at room temperature for 5min. Place it on the magnetic rack, and after the solution is clarified (about 2min), suck out the eluent for standby and measure the concentration.*

*3.2 DNA fragment end repair and purification*

*Prepare the final reaction solution according to Table 6 in a 0.2ml PCR tube, gently stir and mix, 20 ℃ for 5 minutes, 65 ℃ for 5 minutes, 4 ℃ hold*

*Table 6 DNA fragment end repair and purification System*

| *Reagent* | *Volume* |
| --- | --- |
| *H2O* | *Calculate the volume of added DNA and replenish it with water to 12.5ul* |
| *DNA* |  |
| *Terminal modifying Buffer* | *1.75µl* |
| *Terminal modifying enzyme* | *0.75µl* |
| *Total* | 1. *µl* |

*Prepare reaction solution according to Table 7, gently stir and mix, 20 ℃ for 20 minutes, 65 ℃ for 10 minutes, 4 ℃ hold；*

*Table 7. Barcode Connection Reaction System*

| *Reagent* | *Volume* |
| --- | --- |
| *H2O* | *2.5 µl* |
| *erminal modification products* | *5 µl* |
| *Native Barcode* | *2.5 µl* |
| *Connect the reaction liquid* | *10 µl* |
| *Total* | *20 µl* |

*After connecting Barcode, mix all the samples into one tube. Add 1.5 * magnetic beads to the sample tube, blow and mix well, and let it stand at room temperature for 10 minutes (gently stir and mix 2-3 times with your fingers); After the rest is completed, immediately detach and transfer to a magnetic rack. After the solution clarifies (about 5 minutes), discard the supernatant; Remove the sample tube from the magnetic holder and clean it twice with 1.5 * SFB. After each addition of SFB, it should be thoroughly mixed with the magnetic beads, then immediately separated and placed on the magnetic holder. After clarification, discard the liquid. Add 100 µ l of 80% ethanol (do not disturb the magnetic beads), immediately dissociate, remove the remaining ethanol, and dry for 30 seconds; Add 35 µ l of water, blow and mix well, let stand at room temperature for 5 minutes, transfer immediately to a magnetic rack, wait for the solution to clear, transfer for backup, and measure the concentration.*

*3.3 Adapter Mix (AMX) Connection Reaction and Purification*

*Prepare the reaction solution according to Table 8, gently stir and mix, and react at 20 ℃ for 20 minutes；*

*Table 8. Adapter Connection Reaction System*

| *Reagent* | *Volume* |
| --- | --- |
| *Pooled barcoded sample* | *30 µl* |
| *Adapter Mix II (AMII)* | *5 µl* |
| *Ligation buffer* | *10 µl* |
| *ligase* | *5 µl* |
| *Total* | *50 µl* |

*Add 75 to the connecting product after the reaction is completed μ Purify with L magnetic beads, blow and mix well, let stand for 10 minutes, and use 125 μ Clean the L SFB twice, mix it thoroughly with the magnetic beads after each addition of SFB, and then place it on a magnetic rack after instant separation. After clarification, discard the liquid. Immediately after separation, place it on a magnetic rack and apply 10% residual SFB μ L Pipette shall be sucked clean and dried after standing for 30s; Join 12 μ L EB, blow and mix well, let stand for 5 minutes, transfer to a magnetic rack after instant separation, and wait for the solution to clear before measuring the concentration and backup.*

*4. Machine sequencing*

*Melt Sequencing Buffer (SQB), Loading Beads (LB), Flush Tether (FLT), and a tube of Flush buffer (FB) at room temperature. Prepare priming mix in a 1.5 mL centrifuge tube: Take 1 FB and add 30 μ L FLT, shake well and centrifuge briefly. Open the cover of MinION Mk1B and load the chip. Slide the sampling port cover clockwise, open the chip Priming port, set the 1mL Pipette to 180 µ L, insert the suction head of the Pipette into the Priming port, slowly rotate the rotary wheel counterclockwise to make the dial display 200 µ L, or until a small amount of buffer can be seen entering the suction head, and then draw 800 μ The priming mix of L is slowly injected into the flow cell from the Priming port to avoid bubbles, and it is allowed to stand at room temperature for 5 min. Fully mix the loading beads (LB) with a Pipette. In the new EP tube, prepare the sample library as follows: 37.5 µ L Sequencing Buffer (SQB), 25.5 µ L LB, 40-80 ng DNA library, fill with water to 75 µ L, and perform sequencing on the machine.*

1. *Bioinformatics analysis*

*The format of the original data file generated by MinION sequencer is fast5. The MinKnow software is used to complete real-time identification and generate the fastq file, and the MinKnow software is used to filter low quality value sequences（10M）.* *The filtered data was subjected to host DNA removal using Minimap2 2.17-r941(3) software (using the human genome reference sequence Hg38). Multi Sequence alignment of sequencing data and determination of pathogenic microorganisms After data filtering and de host DNA sequencing data, Centrifuge(4) v1.0.3 is used for multi Sequence alignment with NCBI non redundant nucleic acid data (NT) library.*

3. Li H. 2018. Minimap2: pairwise alignment for nucleotide sequences. Bioinformatics 34:3094-3100.

4. Kim D, Song L, Breitwieser FP, Salzberg SL. 2016. Centrifuge: rapid and sensitive classification of metagenomic sequences. Genome Res 26:1721-1729.

***Supplementary 2***

*Table 1. 354 respiratory pathogens were included in the panel of this study*

| *Bacterium (n=236)* | *Gram-positive bacillus* | *Mycobacterium tuberculosis complex group (Mycobacterium tuberculosis, Mycobacterium bovis, Mycobacterium Africana), Mycobacterium leprae, nontuberculous mycobacterium (Mycobacterium avium, Mycobacterium intracellular, Mycobacterium abscess, Mycobacterium Kansus, Mycobacterium Malmo, Mycobacterium toad, Strumfulosa, Mycobacterium chelonis, Mycobacterium fortuitus, Mycobacterium marinum, Mycobacterium gordonum) Bacillus, Mycobacterium myxigenes, Mycobacterium ulcerans, Mycobacterium flavus, Mycobacterium Marseille, Mycobacterium Kumamoto, Mycobacterium Haemophilus, Mycobacterium paracytosus, Mycobacterium Genevae, Mycobacterium Asiatica, Mycobacterium Bohemia, Mycobacterium carinii, Mycobacterium hiddeni, Mycobacterium simiae, Mycobacterium Stefani, Mycobacterium triplica, Mycobacterium bacteremia, Mycobacterium neoaureus, Mycobacterium stigmatis, Mycobacterium Colombia), Nocardia in pus, Nocardia abscess, Nocardia Wallace, star, Nocardia Brasiliensis, Nocardia otitis of guinea pig, Nocardia dermatitis, Nocardia pseudobrasiliensis, Nocardia neocardia, Transsylvania, Nocardia veteranum, Nocardia galsenkirchen, Nocardia brachystreptococcus, Clostridium percapsulatus, Lysogroup Clostridium difficile, Clostridium tetanus, Clostridium botulinum, Clostridium difficile, Clostridium Northrix, Erysipelothrix erysipelogenes, Listeria monocytogenes, Whipples' trophia, Deficient bacteria, Actinomyces madurae, Actinomyces actinoides, Actinomyces caries, Streptomyces, Bacillus anthracis, Bacillus cereus, Clostridium difficile, Clostridium septicus Bacillus, Arcanobacterium pyogenes, Arcanobacterium haemolyticus, Propionibacterium pemphigi, Corynebacterium McCorilli, Corynebacterium diphtheriae, Corynebacterium striatum, Corynebacterium pseudotuberculosis, Rhodococcus equi, Rhodococcus rhodococcus fuchsia* |
| --- | --- | --- |
|  | *Gram-positive coccus* | *Enterococcus faecalis, Enterococcus faecium, Enterococcus aviae, Enterococcus gallinaceus, Enterococcus plumboflavus, Streptococcus agalactis, Streptococcus galactis, Streptococcus pneumoniae, Streptococcus pyogenes, Streptococcus suis, Streptococcus bradystis, Streptococcus cristae, Streptococcus constellatus, anaerobic peptococcus, Streptococcus Donae, Streptococcus gallicae, Streptococcus equi, Streptococcus Miller, Streptococcus bovis, Streptococcus Canis, Angina Streptococcus, Streptococcus ovis, Streptococcus intermedius, Streptococcus piggut, Streptococcus equi, Streptococcus infantile, Streptococcus parahaematococcus, Streptococcus oral, Staphylococcus aureus, Staphylococcus epidermidis, Staphylococcus Luteus, Staphylococcus hominis, Staphylococcus arletus, Staphylococcus chromogenes, Staphylococcus auris, Staphylococcus saprophyticus, Staphylococcus slowus, Staphylococcus pseudointermedius Staphylococcus cocci, Staphylococcus Koch, Staphylococcus Kerri, Staphylococcus equi, Staphylococcus imitatus, Staphylococcus xylosus, Staphylococcus haemolyticus, Staphylococcus goat, Staphylococcus Schisteri, Staphylococcus Sciuris, Staphylococcus capitans, Staphylococcus Woweri, Staphylococcus vealis, Staphylococcus intermedius, Staphylococcus suis, Twinus morbiae, Micrococcus luteus, Cookella roseus, Staphylococcus Streptomyces granulosus and Rhodotella mucilaris* |
|  | *Gram-negative bacilli* | *Acinetobacter Baumannii, Acinetobacter Pitter, Acinetobacter calcic Acetate, Enterobacter sakazakii, Enterobacter cloacae, Proteus mirabilis, Bacillus aureus indolicogenes, Bacillus alcaligenes faecalis, Citrobacter Freudi, Corynebacterium jeri, Bacillus cardiobacterium humani, Bacillus pulmonoides, Bacillus palustris humani, Bacillus rigovitogeni, Bacillus pestis, Bacillus brucei, Bacillus fluoriformis Bozmanni Citrobacter monciformis, Agnetocosa, Citrobacter kirkii, Streptococcus candidiformis, Campylobacter jejuni, Campylobacter foetus, Corynebacterium ureae, Proteus vulgaris, Bordetella pertussis, Bordetella parappertussis, Bordetella avium, Bordetella houthi, Burkholderia melanoides, Burkholderia melanoides, Burkholderia acoris, polyphagosis Burkholderia, Klebsiella aerogenes, Klebsiella acidogenes, Klebsiella pneumoniae, Klebsiella mutans, Escherichia coli, Haemophilus influenzae, Haemophilus parainfluenzae, Haemophilus Haemolyticus, Legionella pneumophila, Pseudomonas aeruginosa, Pseudomonas putidis, Serratia marcescens, Stenotrophomonas maltophilia, Bacillus morgana, Salmonella enterica, Elizabethia meningosepticum , Francisella tulafarensis, Serratia liquefaciens, Pasteurella multocida, Pseudomonas alcaligenes, Kingella kimi, Aeromonas hydrophila, Shigella Sonnei, Bartonella veneri, Bartonella baculosa, Bartonella elizabethica, Elizabethica pacifica, alcaligenes Providencia, Panthenia globosa, Edwardsiella obliqua, Prevotella diplex, Hafniella apiaria, Shiwanella alginae, Vibrio vulnificus, Vibrio cholerae, Rolstonia mannitolytica, Raoulia ornitholytica, Avenella Americana, Rolstonia pieteri, Pseudomonas oryzae, Aikenella erotica, Cynocytophilia Canis, Salmonella typhi, Procencia Steinii, Yersinia enterocolitica* |
|  | *Gram-negative coccus* | *Neisseria meningitidis, Neisseria gonorrhoeae, Neisseria microflava, Neisseria desiccata, Moraxella katamensis, Moraxella Oslo, Moraxella lacunaris* |
|  | *Aanaerobe* | *Bacteroides fragilis, Clostridium nucleatum, Clostridium periodontidis, Clostridium necrosis, Prevotella, Actinomyces, Peptostreptococcus, Peptococcus nigra, Prevotella griseus, Porphyromonas gingivalis, Forsestanella, Veillonococcus atypicus, Veillonococcus parvus, Peptonophilus lacrimalis* |
| *Fungi（n=33）* | | *Aspergillus aflatus, Aspergillus oryzae, Aspergillus fumigatus, Aspergillus nidus, Aspergillus Niger, Aspergillus terreus, Candida albicans, Candida auris, Candida glabrata, Candida Tropicalis, Candida Portuguese, Pichia Kudriazweil (Candida kronensis）*  *Bacillus, Rhizopus oryzae, Sedospora apicum, Fusarium, Blastomyces dermatitis, Sporotrichum Schenckii* |
| *Others*  *(n=18)* | *Mycoplasmas* | *Mycoplasma pneumoniae, Mycoplasma hominis, ureaplasma parvos, Ureaplasma urealyticum* |
|  | *Chlamydia* | *Chlamydia pneumoniae, Chlamydia psittaci, Chlamydia trachomatis* |
|  | *Spirochaeta* | *Syphilis, leptospira* |
|  | *Rickettsiae* | *Rickettsia plati, Rickettsia Mosii, Rickettsia Rickettsia Rickettsia feline, Rickettsia typhus, Orientia tsutschiggers, Benacosia, Hansebartonia, anaplasmosis granulocyticus* |
| *Viruses (n=58)* | *DNA viruses* | *Adenovirus, human adenovirus type B7, human adenovirus type B55, human adenovirus type C1, human adenovirus type C2, human adenovirus type C5, human adenovirus type E4, human adenovirus type B3, human Boca virus, human Parvovirus B19, herpes simplex virus type 1, herpes simplex virus type 2, varicella zoster virus, EB virus (blister type 4) Herpes), Cytomegalovirus (herpes type 5), human herpesvirus type 6, human herpesvirus type 7, human herpesvirus type 8, herpes virus type B, BK polyoma virus, JC polyoma virus, WU polyoma virus, human polyoma virus type 7, KI polyoma virus, human polyoma virus type 5, MW polyoma virus* |
|  | *RNA viruses* | *Influenza A virus H1N1, influenza A virus H3N2, Avian influenza H5N1, Avian influenza H7N9, influenza A virus (universal), influenza B virus, influenza C virus, parainfluenza virus type 1, parainfluenza virus type 2, parainfluenza virus type 3, parainfluenza virus type 4, human metapneumovirus, coronavirus 229E, coronavirus HK U1, Coronavirus NL63, coronavirus OC43, respiratory syncytial Virus A, respiratory syncytial virus B, human rhinovirus, measles virus, rubella virus, mumps virus, Enterovirus 71, Enterovirus C, Enterovirus D, Coxsackie virus A6, Coxsackie virus A16, Coxsackie virus A21, Coxsackie virus B, Echovirus, Rotavirus, novel coronavirus* |
| - *Parasites (n=9)* | | *Paragonimus westermani, Entamoeba histolytica, Echinococcus, Toxoplasma gondii, Microsporidia, Cryptosporidium, Strongyloides stercoralis, Brugia malayi, Toxocara canis* |

***Supplementary 3 CMTs Testing***

Routine microbiological tests were performed on BALF samples. These included Gram staining, acid-fast bacilli smear, bacterial culture, mycobacterial culture, and fungal culture. Additionally, GeneXpert was employed. These tests were complemented by serum G test, GM test, and cryptococcal antigen detection. PCR testing for SARS-CoV-2 and influenza A and B was conducted from throat swabs. Furthermore, Mycoplasma pneumoniae, Chlamydia pneumoniae, respiratory syncytial virus, adenovirus, cytomegalovirus, and Epstein-Barr virus antigen/antibody immunoassays were performed.

Cultivation: According to the "Regulations on Clinical Trial Operation of Pathogen Detection in National Clinical Microbiology Laboratories(5)", all samples underwent routine analysis. Bronchoalveolar lavage fluid (BALF) was concentrated by centrifugation at 3,000 rpm for 10 minutes, and then inoculated onto blood agar plates, MacConkey agar plates, and chocolate agar plates, which were cultured at 35°C with 5% CO2 for 7 days. For suspected mycobacterial cultures, samples were inoculated into Roche medium and cultured for 42 days. Samples from patients suspected of clinical fungal infections were inoculated onto blood agar plates and Sabouraud dextrose agar, and cultured for 7 days. Mass spectrometry analysis (Bruker MBT) was performed within 48 hours after colony growth. Due to limitations in biosafety facilities, viral cultures were not conducted.

Acid-fast bacilli smear: Acid-fast staining was performed using the Ziehl-Neelsen method. Staining analysis was conducted using the BR-101 fully automated tuberculosis-acid-fast staining instrument (Qingdao Lance Biotechnology Co., Ltd.). Subsequently, smear readings were performed using a slide viewer (Ningbo Shunyu automatic smear reader). Positive samples were rechecked at 1000x magnification under an Olympus optical microscope.

Gram staining: Gram staining was performed using the BR-001 fully automated Gram staining instrument (Qingdao Lance Biotechnology Co., Ltd.). Manual slide reading was conducted using an Olympus optical microscope.

GeneXpert: Mix the centrifuged lavage fluid sample with 2 mL of sample processing solution. After vortexing for 15 seconds, incubate the mixture at room temperature for 15 minutes. Then, aspirate the liquefied sample into the dedicated sterile transfer pipette of the Xpert MTB/RIF test cartridge; open the test cartridge and slowly add the processed sample into the sample well of the cartridge, close the test cartridge for automated testing. The system automatically reads the MTB test results within 2 hours (Xpert MTB/RIF semi-nested fully automated real-time fluorescence quantitative PCR detection technology 301-3300-ZH-CN).

PCR testing for COVID-19 and influenza A/B: Patient throat swab samples were subjected to PCR testing for COVID-19, influenza A, and influenza B using the Roche 480 fluorescence quantitative PCR instrument.

Serum Antibodies: Serum samples were sent to Hangzhou Di'an Digital Diagnostic Laboratory for detection of antibodies against Mycoplasma pneumoniae, Chlamydophila pneumoniae, respiratory syncytial virus, adenovirus, cytomegalovirus, and Epstein-Barr virus.

G and GM Assays: Serum samples were sent to Hangzhou Di'an Digital Diagnostic Laboratory for detection of fungal antigens including (1,3)-β-D-glucan and galactomannan.

Cryptococcal Antigen (Latex Agglutination): One drop of serum sample handling solution was added to an EP tube, followed by the addition of 40μl of the sample. After thorough mixing, a cryptococcal capsular polysaccharide detection strip (IMMY Cryptococcal Antigen LFA) was directly immersed into the diluted specimen. The result was determined after ten minutes.

1. Gaston DC, Miller HB, Fissel JA, Jacobs E, Gough E, Wu J, Klein EY, Carroll KC, Simner PJ. 2022. Evaluation of Metagenomic and Targeted Next-Generation Sequencing Workflows for Detection of Respiratory Pathogens from Bronchoalveolar Lavage Fluid Specimens. J Clin Microbiol 60:e0052622.

2. Li S, Tong J, Liu Y, Shen W, Hu P. 2022. Targeted next generation sequencing is comparable with metagenomic next generation sequencing in adults with pneumonia for pathogenic microorganism detection. J Infect 85:e127-e129.

3. Li H. 2018. Minimap2: pairwise alignment for nucleotide sequences. Bioinformatics 34:3094-3100.

4. Kim D, Song L, Breitwieser FP, Salzberg SL. 2016. Centrifuge: rapid and sensitive classification of metagenomic sequences. Genome Res 26:1721-1729.

5. Shang H, Mao Y, Ding J, Ren J, Zhou W. 2014. Guideline for clinical laboratory biosafety(WS/T 442-2014). National Health and Family Planning Commission,People's Republic of China http://www.nhc.gov.cn/ewebeditor/uploadfile/2014/07/20140731165354106.PDF.

***Supplementary 4 Culturable pathogens were detected in this study.***

Specific types of culturable pathogens defined in the tNGS group.

|  | Pathogens |
| --- | --- |
| Easy to Cultivate | Pseudomonas aeruginosa, Klebsiella aerogenes, Enterococcus faecalis, Acinetobacter baumannii, Staphylococcus aureus, Enterobacter cloacae |
| Fastidious bacteria | Haemophilus influenzae, Moraxella catarrata, Streptococcus pneumoniae |
| Mycobacterium | Mycobacterium tuberculosis, Mycobacterium intracellular, nontuberculous Mycobacterium |
| Fungi | Aspergillus, candida, Cryptococcus |

Specific types of culturable pathogens defined in the NTS group.

|  | Pathogens |
| --- | --- |
| Easy to Cultivate | Stenotrophomonas maltophilia, Klebsiella pneumoniae, Pseudomonas aeruginosa, Staphylococcus aureus, Nocardia, Acinetobacter baumannii, Citrobacter, Streptococcus constellatus |
| Fastidious bacteria | Haemophilus influenzae, Bordetella pertussis, Streptococcus pneumoniae, Moraxella catarrata |
| Mycobacterium | Mycobacterium intracellular, Mycobacterium tuberculosis, Mycobacterium kansensis, Mycobacterium abscessus, Mycobacterium avium complex group |
| Fungi | Aspergillus, candida, Cryptococcus |
